# A systematic review and meta-analysis of bone loss in space travelers

**DOI:** 10.1038/s41526-020-0103-2

**Published:** 2020-05-05

**Authors:** Mariya Stavnichuk, Nicholas Mikolajewicz, Tatsuya Corlett, Martin Morris, Svetlana V. Komarova

**Affiliations:** 1 Department of Biomedical Engineering, McGill University, Montréal, Canada; 2 Shriners Hospital for Children—Canada, Montréal, Canada; 3 Faculty of Dentistry, McGill University, Montréal, Canada; 4 Schulich Library of Physical Sciences, Life Sciences and Engineering, McGill University, Montréal, Canada

**Keywords:** Physiology, Medical research

## Abstract

Bone loss in space travelers is a major challenge for long-duration space exploration. To quantify microgravity-induced bone loss in humans, we performed a meta-analysis of studies systematically identified from searching Medline, Embase, Web of Science, BIOSIS, NASA Technical reports, and HathiTrust, with the last update in November 2019. From 25 articles selected to minimize the overlap between reported populations, we extracted post-flight bone density values for 148 individuals, and in-flight and post-flight biochemical bone marker values for 124 individuals. A percentage difference in bone density relative to pre-flight was positive in the skull, +2.2% [95% confidence interval: +1.1, +3.3]; neutral in the thorax/upper limbs, −0.7% [−1.3, −0.2]; and negative in the lumbar spine/pelvis, −6.2 [−6.7, −5.6], and lower limbs, −5.4% [−6.0, −4.9]. In the lower limb region, the rate of bone loss was −0.8% [−1.1, −0.5] per month. Bone resorption markers increased hyperbolically with a time to half-max of 11 days [9, 13] and plateaued at 113% [108, 117] above pre-flight levels. Bone formation markers remained unchanged during the first 30 days and increased thereafter at 7% [5, 10] per month. Upon landing, resorption markers decreased to pre-flight levels at an exponential rate that was faster after longer flights, while formation markers increased linearly at 84% [39, 129] per month for 3–5 months post-flight. Microgravity-induced bone changes depend on the skeletal-site position relative to the gravitational vector. Post-flight recovery depends on spaceflight duration and is limited to a short post-flight period during which bone formation exceeds resorption.

## Introduction

Since the mid-seventies, space travelers have been known to experience severe bone loss at a rate of 1–1.5% per month, which is only partially responsive to non-pharmacological countermeasures^1,2^. Pharmacological treatments, such as anti-resorptive bisphosphonates, reduce bone loss in-flight, but may interfere with the slow and often incomplete post-flight recovery^2^. Thus, microgravity-induced bone loss is a significant and unresolved health risk for space travelers.

Bones support body weight and transmit forces generated by muscles, adapting to endure mechanical loads^3^. The skeleton also serves as a mineral reservoir^4^, accommodates hematopoietic bone marrow^5^, and plays an active role in acid–base homeostasis^6^. Since many of these functions are affected by microgravity, including reduced mechanical loading^7^, altered calcium homeostasis^8^, reduced hematopoiesis^9^, and altered metabolism^10^, the relative contributions of different processes to bone loss in space remain unresolved. Bone health is assessed using imaging radiography, a technique that over time has developed from projection radiography, through single photon absorptiometry (SPA), to dual X-ray absorptiometry (DXA) and quantitative computed tomography (qCT) now widely used in a clinical setting^11^. All these methods were used at some time to assess bone health in space travelers, however, due to the absence of radiographic equipment aboard spacecraft, all bone density measurements were acquired on Earth, immediately before and after a spaceflight.

Bone adaptation requires the actions of bone cells: bone-forming osteoblasts and bone-destroying osteoclasts. Bone formation and resorption can be approximated from the biochemical by-products of osteoblast and osteoclast function. Osteoblasts secrete bone specific alkaline phosphatase (BSAP) and osteocalcin (OC), and produce a collagen type I-based organic matrix, which is coupled with cleavage of C- and N-terminal propeptides of collagen type I (PICP and PINP)^12^. Tissue-nonspecific alkaline phosphatase (AP) is also used as a bone formation marker^12^. During bone resorption, osteoclasts degrade organic matrix, releasing amino acids such as hydroxyproline (HP), fragments of collagen type I, including C- and N-terminal telopeptides (CTX and NTX), as well as pyridinoline (PYD) and deoxypyridinoline (DPD)^12,13^. The biochemical markers produced by osteoblasts and osteoclasts are measured in the urine and serum to estimate bone turnover during spaceflight.

The science of space exploration is challenging and costly from a technological and medical perspective. Small teams of individuals participate in missions of different duration in spacecraft that change dramatically with time. Thus, obtaining statistical power that is sufficient to discern biological effects from random variation is a prevalent challenge. Many studies have reported that humans lost bone during spaceflight^1,2^; however, it is difficult to find data related to (i) changes in different skeletal regions, (ii) temporal kinetics of bone loss, (iii) relationship between bone and bone cell function, and (iv) degree of individual variability. With the objective to estimate these parameters, we used a meta-analytic approach to combine systematically identified data reporting measurements of bone density or biochemical bone markers in humans who have been to space according to the Fédération Aéronautique Internationale (FAI) definition. In the rest of the manuscript, we will use the term “astronaut” to define any person who traveled to space according to the FAI definition independent of their country of origin.

## Results

### Publications on bone health in astronauts

#### Article identification

**Fig. 1:**
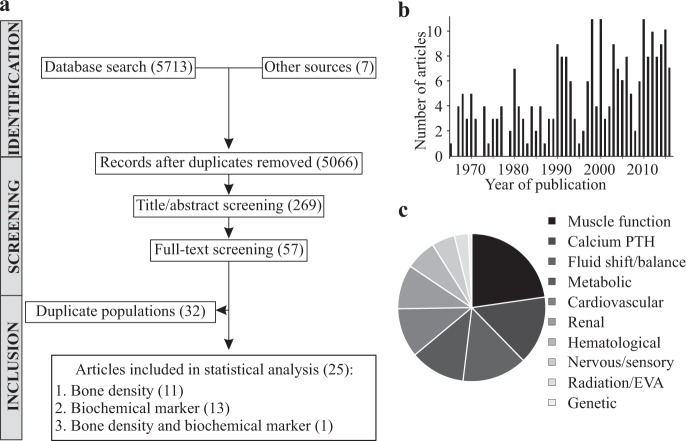
Systematic review information flow and outcomes. **a** Prisma diagram. **b** Number of relevant articles by publication year. **c** Physiological processes suggested to contribute to bone loss in space.

The systematic search in Medline, Embase, Web of Science, and BIOSIS databases identified 5713 candidate articles related to bone health in humans who traveled to space (Fig. 1a). Seven additional reports were found in the NASA technical report server database. After title/abstract screening, we identified 269 articles relevant to bone health in astronauts (Fig. 1a, b). Physiological factors identified as relevant to bone health in astronauts included muscle function, calcium homeostasis, fluid shift, metabolic, cardiovascular, and renal functions (Fig. 1c). After full-text screening, we identified 57 manuscripts which reported numerical data on changes in bone-related outcomes during or after spaceflight.

#### Astronaut identification

To avoid duplicate datasets in the meta-analysis, we attempted to identify astronauts in each study and found a number of studies that reported the findings for the same astronauts. When two articles reported data for the overlapping astronaut populations, we included: (i) both studies if different outcomes were reported, (ii) the study reporting the more complete dataset for overlapping reported outcomes, or (iii) the study with a higher quality score for the same reported outcomes. We could not ensure the absence of overlap between two studies, therefore data for five astronauts may have been included twice in the analysis^1,14^.

#### Articles included for meta-analysis

We selected 25 articles for meta-analysis, including 12 studies that reported bone density measures before and less than a week after a spaceflight^1,14–23^, 14 studies that contained data on biochemical bone markers^24–36^, and one study that reported both^37^. The final dataset contained data for ~189 astronauts (the number is approximate due to remaining uncertainty in astronaut identification), with bone density measurements and biochemical bone markers available for ~148 and ~124 astronauts, respectively.

### Changes in bone density during spaceflight

#### Skeletal site-specific changes in bone density

**Fig. 2:**
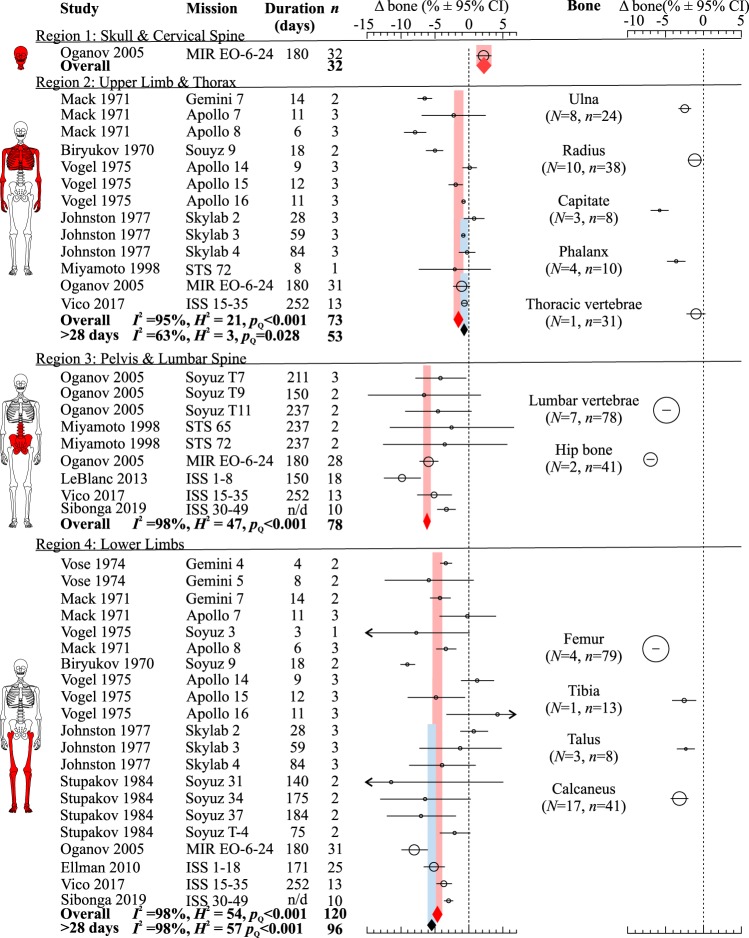
Spaceflight-related bone density changes in different skeletal regions. Forest plots of changes (Δ) in bone density (% of pre-flight) in the skull, cervical vertebrae (region 1); upper limbs, thoracic vertebrae, ribs (region 2); pelvis, lumbar vertebrae (region 3); and lower limbs (region 4) (left); and in individual bones (right). Circles/lines: effect sizes (marker sizes are proportional to number of astronauts) and 95% CI; red diamonds/bands: overall effects ± 95% CI; blue diamonds/bands: overall effects ± 95% CI for data from missions longer than 28 days. Dashed line: no change from pre-flight. N/d: not determined. Source papers are in mission order. Missions, their duration, number of missions/aggregated missions (*N*), and sample sizes (*n*) are shown.

We examined changes in bone density in four skeletal regions: skull and neck (region 1), upper limbs and thoracic vertebrae (region 2), pelvis and lumbar vertebrae (region 3), and lower limbs (region 4) (Fig. 2 and Supplementary Table 1). Spaceflight resulted in significant bone gain in the skull region 2.2% [1.1, 3.3] and significant bone loss in the thorax and upper limbs −1.4% [−2.1, −0.6], lumbar spine/pelvis −6.2% [−6.7, −5.6], and lower limbs −4.9% [−5.6, −4.2]. The trends of bone density changes in each region were consistent with changes in individual bones within each region (Fig. 2 and Supplementary Table 2). Very short missions are likely of insufficient duration to accurately detect changes in bone density^38^. Therefore, we estimated bone density changes after spaceflights longer than 28 days in region 2, where an updated value was less different from baseline, −0.7% [−1.3, −0.2] and region 4, where the new estimate indicated more severe bone loss −5.4% [−6.0, −4.9]. Coefficient of variation, which indicates relative variability of the measure, was higher for regions 1 (26%) and 2 (19%) compared to regions 3 (5%) and 4 (7%).

#### Heterogeneity and bias

Statistical heterogeneity accounted for >95% of the total variance in bone density data (Fig. 2). The meta-analytic outcomes were not significantly influenced by study quality, year of publication, or any single dataset (Supplementary Fig. 1b, c). After ~20% of most heterogeneous studies were removed, the homogenous datasets reported lower bone loss in the upper limb and thorax region, but not in the lower limb region (Supplementary Fig. 1d, e). Funnel plot analysis suggested an underreporting of positive bone density changes in region 2.

**Fig. 3:**
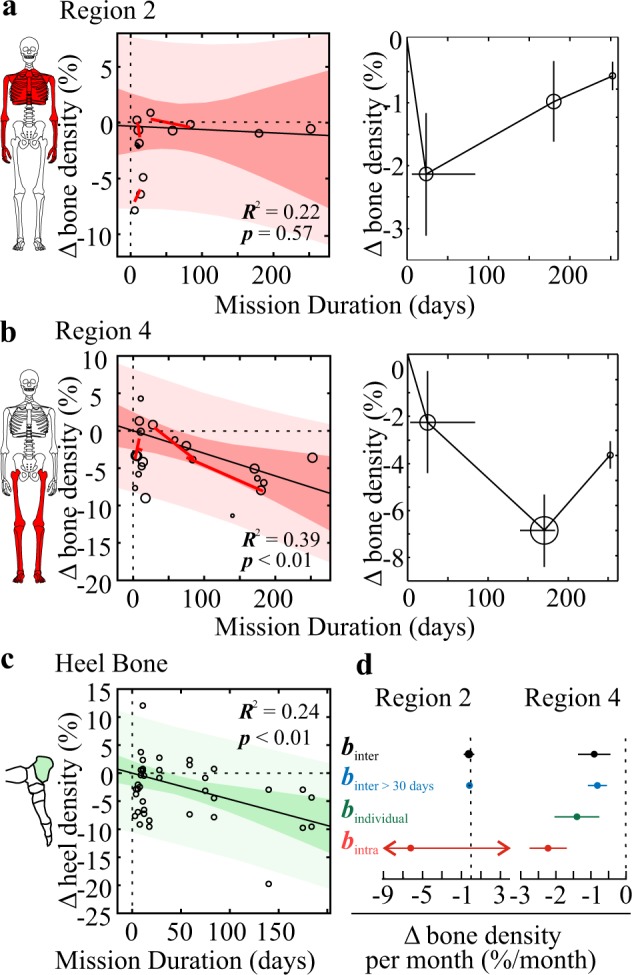
Changes in bone density as a function of mission duration. **a**–**c** Effect of space flight duration on changes in bone density (% of pre-flight) in region 2 (upper body, **a**), region 4 (lower limbs, **b**), and heel bone of individual astronauts (**c**) was assessed by meta-regression (left for (**a**–**c**)) and subgroup analysis (right for **a**, **b**). For meta-regression, black solid line/red bands: inter-study (meta) regression ± 95% confidence (dark red) and prediction (light red) intervals; red lines: intra-study regressions. For subgroup analyses, mission-level changes were pooled by mission duration (<100, 100–200, >200 days) and plotted as a function of average mission duration. Horizontal error bars: range of mission durations within subgroup; vertical error bars: pooled standard errors. Marker sizes are proportional to number of astronauts. **d** Rate of bone loss for regions 2 (left) and 4 (right): slope coefficients *β* ± 95% CI for meta regressions for all mission durations (*β*_inter_, black), missions longer than 30 days (*β*_inter_ > 30 days, blue), individual heel bone data (*β*_individual_, green), and average intra-study regressions (*β*_intra_, red). For region 2, *β*_intra_ = −6 [−21, 9].

Temporal changes in bone density were examined using meta-regression and subgroup analysis for short (<100 days), intermediate (100–200 days), and long (>200 days) missions (Fig. 3). In region 2, meta-regression reported no relationship between bone density changes and mission duration, while subgroup analysis demonstrated that highest bone loss was reported in short missions (Fig. 3a). In contrast, changes in lower limb bone density were strongly associated with mission duration by meta-regression (*p* < 0.01) and subgroup analysis (Fig. 3b). Consistently, changes in individual heel bone density were also significantly associated with mission duration (*p* < 0.01) (Fig. 3c). For both regions 2 and 4, the rates of bone density change estimated from within-study regressions were higher and more variable compared to meta-analytic results (Fig. 3d). For the region 4, the rates of bone loss were similar for all missions, missions longer than 30 days, and heel bone estimates (Fig. 3d). The most conservative and precise estimates for the rate of bone loss were obtained for missions longer than 30 days, which were −0.1% [−0.2, 0.0] per month for upper limbs and thorax, and −0.8% [−1.1, −0.5] per month for lower limbs. Coefficient of variation for the rate of bone loss in region 4 was similar for the aggregate (26%) and individual heel bone (23%) estimates.

### Changes in biochemical bone markers during and after spaceflight

#### Agreement between biochemical markers

Pair-wise correlation analysis for biochemical bone markers measured in serum (s) or urine (u) demonstrated consistent changes for the markers of bone resorption uHP, uNTX, uDPD, and uCTX; and formation sBSAP, sAP, and sP1CP, while uPYD and sOC correlated poorly with other biochemical markers (Supplementary Figs. 2 and 3). Different resorption and formation markers were pooled together for subsequent analysis.

#### In-flight changes in biochemical markers

**Fig. 4:**
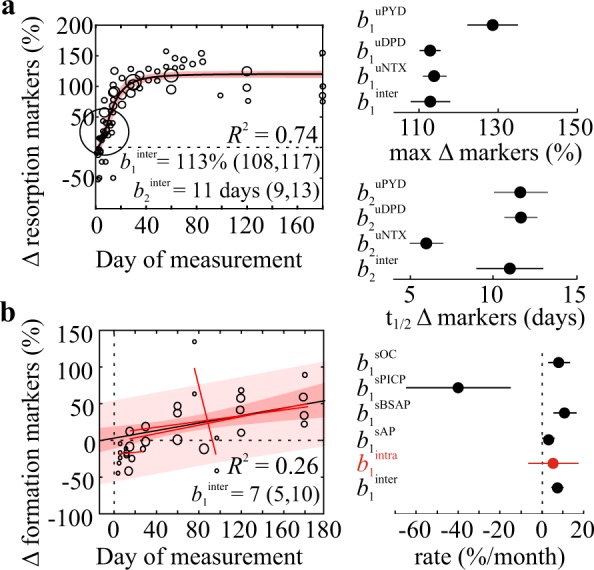
Changes in biochemical bone markers during spaceflight. **a** The effect of flight duration on resorption markers (% pre-flight) was modeled using a sigmoidal function. Left: circles are study-level changes, marker sizes are proportional to number of astronauts, black line/red band: mean fit/95% CI. Right: max levels (*β*_1_) and time to half-max (*β*_2_) with 95% CI for pooled (*β*_inter_) and individual (*β*_uPYD_, *β*_uDPD_, *β*_uNTX_) markers. **b** The effect of flight duration on formation markers (% pre-flight) was assessed by meta-regression. Left: circles are study-level changes, marker sizes proportional to number of astronauts, black line, dark/light red bands: meta-regression with 95% confidence/prediction intervals, red lines: intra-study regressions. Right: rates of change (*β*_1_) ± 95% CI for pooled (*β*_inter_, *β*_intra_) and individual (*β*_sOC_, *β*_sPICP_, *β*_sBSAP_, *β*_sAP_) markers.

In-flight, bone resorption markers increased with a half-time to maximum of 11 [9, 13] days to 113% [108, 117] above pre-flight levels (Fig. 4a, left). The rate of increase for uDPD and uPYD was consistent with overall estimates, while uNTX increased significantly faster with a half-time of 6 [5, 7] days (Fig. 4a, right). Bone formation markers demonstrated a weak positive association (*R*^2^ = 0.26, *p* < 0.001) with time in-flight (Fig. 4b, left). The linear rate of formation markers increase was 7% [5, 10] per month, which was consistent with estimates from single studies, and for individual markers except for sPICP (Fig. 4b, right). Coefficients of variation were 9% for a half-time and 2% for maximal levels for bone resorption markers, and 15% for formation markers.

#### Post-flight changes in biochemical markers

**Fig. 5:**
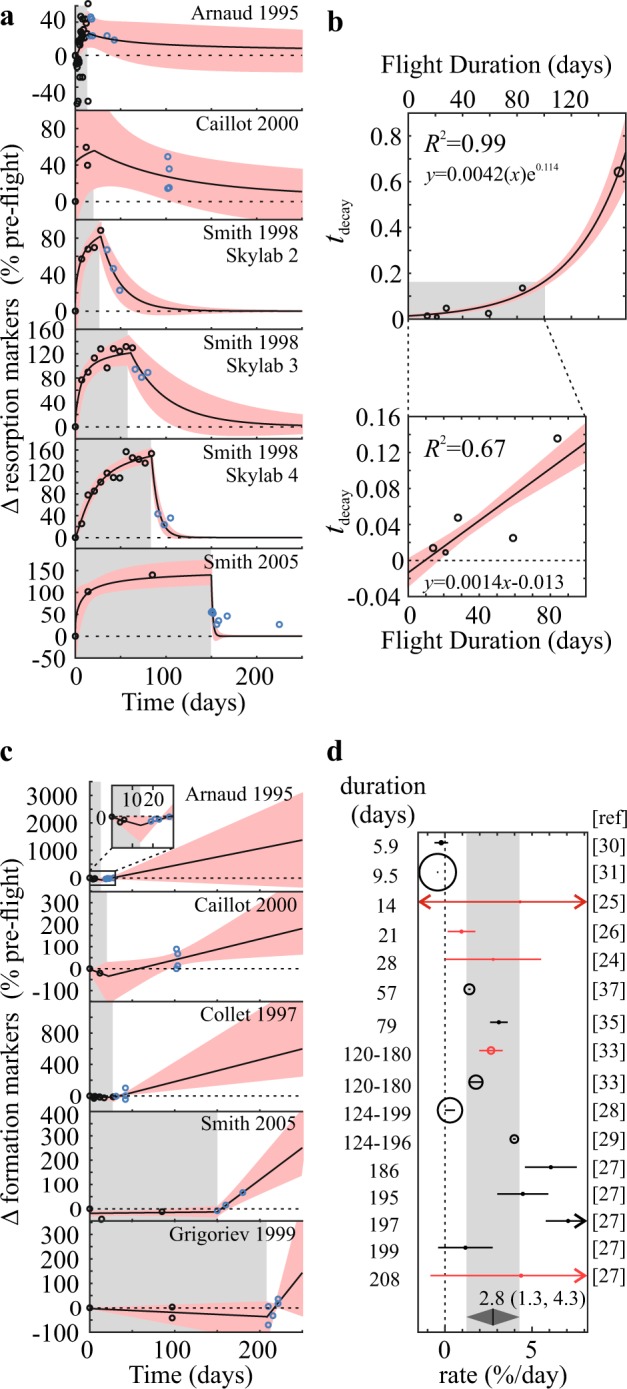
Post-flight changes in biochemical bone markers. **a**, **c** Changes in markers of resorption (**a**) and formation (**c**) (% of pre-flight) were extracted from the studies that reported both in-flight and post-flight measurements and fit to a piece-wise function: **a** sigmoidal in-flight, exponential post-flight; **c** linear in-flight and post-flight. Black line/red band: mean fit/95% CI, studies are arranged in order of mission duration (gray background). **b** The effect of spaceflight duration on post-flight decay constant (*τ*_decay_) of resorption markers was modeled using exponential function for all flight durations (top) or linear function for mission durations <90 days (bottom). Black line/red band: mean fit/95% CI. **d** Forest plot of rates of post-flight changes in formation markers sorted by mission duration. Red circles: studies with in- and post-flight data, black circles—studies with only post-flight data. Gray diamond/band: overall estimate ± 95% CI.

The starting point for post-flight recovery depends on how much biochemical markers changed in-flight, which in turn depends on flight duration. To address this, we used a subset of studies reporting both in- and post-flight changes in biochemical markers, which were fit to piece-wise functions using the Monte-Carlo method (Fig. 5). In-flight changes in resorption markers were modeled with a sigmoidal function, and post-flight changes with an exponential function forced through the last in-flight value. Resorption markers consistently decreased to pre-flight levels at an exponential rate (Fig. 5a), however, the rate of decay was faster in individuals who participated in longer flights (Fig. 5b). In- and post-flight changes in formation markers were fit with linear functions (Fig. 5c). While the complete in-flight formation marker dataset suggested that bone formation increased in-flight (Fig. 4b), mission-level datasets suggest that it remained unchanged or slightly decreased in-flight (Fig. 5c). Upon return to Earth, bone formation markers increased linearly (Fig. 5c) with an overall rate of 2.8% [1.3, 4.3] per day or 84% [39, 129] per month (Fig. 5d). The reported rates of change were highly variable between studies, ranging from −12.0 to 213% per month. Only two studies reported bone formation markers later than 30 days after landing. Caillot-Augusseau and colleagues reported that in one astronaut from 1995 to 1997 Mir missions undercarboxylated osteocalcin was still elevated 80 days post-flight^26^. Smith and colleagues reported that in 12 astronauts from Shuttle-Mir program bone formation markers returned to baseline by 150 days post-flight^33^.

### Potential mediators of spaceflight-related bone loss

**Fig. 6:**
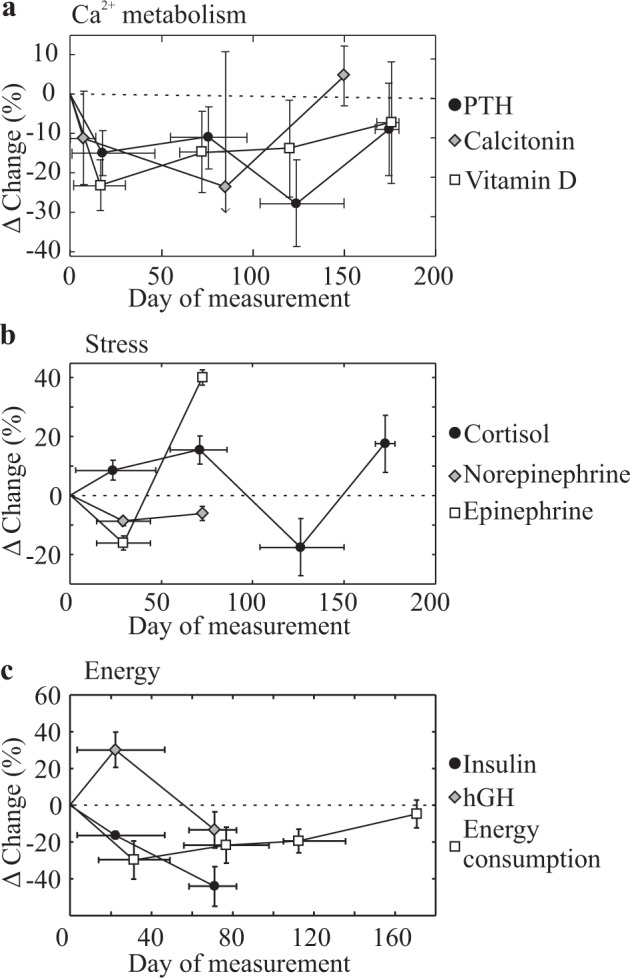
Spaceflight-related changes in physiological factors potentially contributing to bone health. Subgroup analyses of space-flight-related changes (% pre-flight) in **a** regulators of calcium metabolism: PTH (*n* = 15–30 astronauts), calcitonin (*n* = 5–17), and vitamin D (*n* = 12–27); **b** stress regulators: cortisol (*n* = 1–7), norepinephrine (*n* = 9) and epinephrine (*n* = 9); and **c** parameters related to energy metabolism: insulin (*n* = 3–6), growth hormone (*n* = 3–6), and energy consumption (*n* = 25–29). Data are means ± SEM grouped by mission duration (<50, 50–100, 100–150, >150 days) with horizontal error bars indicating the range of mission durations within a subgroup.

We explored the availability of quantitative data for potential mediators of bone loss using the library of 269 papers selected for full-text screening. We identified studies that reported in-flight changes in regulators of Ca^2+^ homeostasis^16,25,27,33,34,37,39^, stress^37,39^, and energy homeostasis^40,41^. Calcium regulating hormones, parathyroid hormone (PTH), 1,25-dihydroxyvitamin D, and calcitonin, were decreased by 11–23% early in spaceflight and gradually returned to pre-flight values thereafter (Fig. 6a). In-flight changes in stress hormones, cortisol, epinephrine, and norepinephrine were variable (Fig. 6b). Energy consumption decreased in the first 30 days of spaceflight and slowly returned to baseline by ~160 days, insulin levels decreased over 80 days in-flight, while growth hormone transiently increased early in-flight (Fig. 6c). The kinetics of changes in calcium regulating hormones and energy consumption were alike to those of formation markers, while none of the potential mediators behaved similar to resorption markers.

### Using meta-analysis to plan future space-flight studies

We used meta-analytic variance estimates to calculate sample sizes required to detect expected spaceflight-related changes (% from pre-flight) in bone density, resorption markers, and formation markers with an 80% power at a 95% significance level. To detect spaceflight-related change in bone density, 10–20 astronauts and >2 months are required; to detect changes in resorption markers, 5–10 astronauts and 0.5–1 month are required; to detect changes in formation markers, 10–20 astronauts and >4 months are required. Appropriately powering investigation of specific countermeasures that target bone resorption or formation will allow not only to draw rigorous conclusions, but also to identify individuals particularly protected or prone to the microgravity-induced bone loss.

## Discussion

We systematically reviewed and quantitatively synthesized published literature on bone health in astronauts. Spaceflight-related changes in bone density were skeletal-site-dependent, with bone gain reported in the skull and cervical vertebrae, no change in the thorax and upper limbs, and progressive bone loss in lumbar spines, pelvis, and lower limbs. Biochemical markers of bone resorption increased robustly within 11 [9,13] days to 113% [108, 117] above pre-flight levels, while bone formation markers increased slowly at a rate of 6% [5, 7] per month. Post-flight, resorption markers decreased exponentially at a rate that was faster after longer duration missions, while formation markers increased linearly at 84% [39, 129] per month. Changes in bone resorption markers were the most consistent among individuals (coefficient of variation 2–9%), while individual variability was higher for bone formation markers (coefficient of variation 15%) and for the rate of bone loss in lower limbs (coefficient of variation 26%). Quantitative estimates of spaceflight-related changes in bone health provided by our study will inform future studies and allow to generate novel hypotheses regarding the underlying mechanisms of observed effects.

The meta-analytic estimate for the rate of bone loss of −0.8% [−1.1, −0.5] per month in the lower limbs region is consistent with previous estimates of 1.0–1.5% decrease per month^42^. We have found that bone is preserved in the upper skeleton and is lost in the lower skeleton, thus corroborating the association between bone density changes and skeletal site position relative to the gravitational vector proposed by Oganov and colleagues^19^. These data, as well as reported differences in trabecular and cortical bone^1,42^, suggest that local factors, such as mechanical environment, or fluid redistribution^43,44^, are important determinants of bone loss; or that bone cells sensitivity to systemic factors depends on skeletal location and/or type^45^. These findings are also important for the interpretation of biochemical bone markers data that reflect bone turnover in the entire skeleton, which has opposing tendencies in different skeletal regions.

Bone loss in the lower limbs was progressive; however, long-duration missions reported less bone loss than intermediate duration missions, suggesting that microgravity-induced bone loss may diminish with time. Consistent with this notion, resorption markers increased rapidly and plateaued after ~25 days in-flight, while formation markers increased slowly, yet continuously, so that the ratio of resorption to formation appeared to gradually reverse from favoring bone loss early in flight to favoring bone formation later. However, this optimistic interpretation should be cautioned by the following considerations: (i) individual-level heel bone loss was proportional to flight durations; (ii) the highest individual bone loss was reported after an intermediate duration flight, likely overestimating bone loss in this subgroup; (iii) increase in bone formation markers was highly variable and meta-analytic estimates differed from individual studies; (iv) longer duration missions included ISS flights that benefited from advanced nutrition and exercise^46^. It is also of interest to consider whether consistent changes in bone resorption (coefficient of variation 2–9%) are directly driven by microgravity, while more variable changes in bone formation (coefficient of variation 15%) are affected by individual’s covariates (i.e., age, physical activity, nutrition, etc.). More data from longer-duration spaceflights are required to test these hypotheses.

Although we limited our analyses to changes in bone density measured immediately post-flight, several studies reported that 2–5 years are required to recover microgravity-induced bone loss^2,19,47^, and that in some individuals the complete recovery was not achieved^2^. We used the studies that reported both in-flight and post-flight changes in biochemical markers to account for the mission duration-dependent in-flight changes in bone markers. Consistent with study-level findings^1,24,26,32,33,39^, meta-analysis demonstrated that post-flight resorption markers quickly declined, while formation markers increased linearly. Surprisingly, following longer duration flights, resorption markers returned to baseline significantly faster than after shorter flights, while changes in formation markers were minimally associated with flight duration. Nevertheless, based on previous studies^26,32^, the active recovery phase, when bone resorption was suppressed and bone formation was active, appears to be limited to 6 months post-flight, much shorter than the time required for bone mass to return to pre-flight values^2^.

Lack of mechanical loading has long been speculated to cause bone loss in microgravity. However, several lines of evidence suggest that it is either not the sole factor, or that the effects of unloading do not comply with the Frost’s mechanostat theory^48^. First, exercise regimes only partially protected against bone loss^46^. Second, bone gain was observed in the skull, which is mechanically neutral. Finally, the mechanostat theory postulates that unloading-induced bone loss is adaptive, implying that after strain is normalized by bone loss, the signal to induce bone resorption should diminish. However, we found no evidence of temporal adaptation of resorption markers. These data suggest a contribution of additional mediators to bone loss in microgravity. Over the 50 years of space travel, many factors, including altered calcium homeostasis^8^, stress^49^, altered metabolism^50^, and radiation^51^ have been suggested to contribute to bone loss in astronauts. We suggest that the kinetics of microgravity-induced changes in potential mediators can be used to implicate them in changes in bone resorption (factors that demonstrate fast switch to a new steady state) or bone formation (factors that change slowly with opposing trends during the initial and late stages of spaceflight). Preliminary estimates suggest that changes in regulators of calcium homeostasis and energy intake have similar dynamic trends as formation markers, but none of the factors behaved similarly to resorption markers. Although no causative conclusions can be derived from these data, such analyses will allow future studies to focus on more promising putative mediators.

The limitations related to the secondary analysis of published data were inconsistent reporting and difficulty in unique identification of astronauts in recent publications. While this is commendable with respect to patient confidentiality and ethical reporting of medical data^52^, we could not ensure that the data for five astronauts were not included twice, and were limited in probing individual-level covariates. The limitations related to technical and biological factors included high variability in outcomes reported for short duration missions, and inconsistency in some markers of bone turnover. To ensure the study validity, we conducted a comprehensive panel of diagnostic tests (single- and cumulative-study exclusion and funnel plot analyses) that demonstrated that our estimates of bone loss in the lower limb region were robust. Since drastic changes in bone mass over 6–16 days missions are physiologically unfeasible^53^ and errors have been reported in early flight bone measurements^23^, we believe that the estimates derived from flights longer than 30 days are more accurate.

In summary, we have conducted a systematic quantitative review of bone health-related changes in astronauts who participated in the Gemini, Apollo, Soyuz, Skylab, Salyut, STS, Mir, and ISS missions. We demonstrate that microgravity-induced changes in bone density depend on the position of the skeletal-site relative to the gravitational vector, provide evidence that bone loss may diminish during longer duration flights, and reveal that post-flight bone recovery depends on the duration of the spaceflight but is limited by a relatively short phase during which bone formation exceeds resorption. Our study was limited by data availability (~189 out of 565 astronauts), inconsistent reporting, and incomplete information provided by certain studies—the limitations reported by other systematic reviews of spaceflight-related health outcomes^54,55^. The analyses conducted in the current study are invaluable for the design of future spaceflight studies and identification of potential study challenges, as demonstrated by our sample size calculations. Moreover, we demonstrated the feasibility of exploratory studies using prior literature to advance new concepts in understanding mechanisms responsible for bone density changes observed in astronauts, which is imperative for a design of successful countermeasures.

## Methods

This study was compliant with the Preferred Reporting Items for Systematic Reviews and Meta-analysis (PRISMA) statement^56^.

### Information sources, search strategy, quality assessment

A systematic search strategy that included the concepts of bones, bone health, terms related to space travel, and the specific names of astronauts, missions, and spacecraft was constructed by a medical librarian (MM) for Ovid Medline (Supplementary Methods 1), translated to Embase (via Ovid), Web of Science, and BIOSIS Previews, and executed on November 21, 2017. An update was performed on Medline and Embase on November 1, 2019. NASA Technical report server and HathiTrust Digital Library were searched for titles of missions and programs. Title/abstract screening was conducted by two independent reviewers (S.V.K. and M.S.). Articles were included for full-text analysis if abstracts indicated reporting quantitative data for bone density or biochemical bone markers in humans during and/or after spaceflight. The eligible studies were scored for the reporting quality (Supplementary Methods 2).

### Data extraction

Data extracted by M.S. and reviewed by T.C. included name and duration of mission; number of astronauts; individual, mean or median percentage changes in bone density or biochemical markers compared to pre-flight; pre-flight, in-flight, or post-flight levels of biochemical markers; standard deviations, standard errors of the mean, and/or interquartile ranges; day or range of days when measurements were performed. If the type of measure of the dispersion was not stated, it was assumed to be a standard error, which ensures a conservative estimate. If a range of sample sizes was reported, the smallest value was extracted. Data from graphs were extracted using MetaLab^57^.

### Study-level outcomes

Outcomes for individuals or groups of astronauts who participated in the same mission were extracted or calculated as percentage from pre-flight $\bar \theta _i = \frac{{\left( {\theta _{\mathrm{x}} - \theta _{{\mathrm{pre}}}} \right)}}{{\theta _{{\mathrm{pre}}}}} \times 100\%$ with standard deviations ${\mathrm{SD}}_{i} = \sqrt {\frac{{\left( {\frac{{100{\mathrm{\% }} \times {\mathrm{SD}}_{{\mathrm{pre}}}}}{{\theta _{{\mathrm{pre}}}}}} \right)^2}}{{n_{{\mathrm{pre}}}}} + \frac{{\left( {\frac{{100{\mathrm{\% }} \times {\mathrm{SD}}_{x}}}{{\theta _{x}}}} \right)^2}}{{n_{x}}}}$, where *x* is in- or post-flight data. When medians $\mathop {\theta }\limits^ {\check{}} _i$ and interquartile ranges *b*_*i*_–*a*_*i*_ were reported, we approximated $\bar \theta _i = \frac{{a_i + \mathop {\theta }\limits^ {\check{}} _i + b_i}}{3}$ and ${\mathrm{SD}}_i = \frac{{b_i - a_i}}{{\eta \left( n \right)}}$, where $\eta \left( {n} \right) = 2E\left( {Z_{\left( {\left. {3n + 1} \right)/4} \right)}} \right)$, and *E*(*Z*_(*n*)_) is the value of order statistic of a random variable *Z*_(*n*)_^58^. Mission-level standard errors were computed as ${\mathrm{SE}}_i = \frac{{{\mathrm{SD}}_i}}{{\sqrt {n_i} }}$, where *n*_*i*_ is the mission sample size.

### Data preparation prior to meta-analysis

To ensure statistical independence, the outcomes measured using different methods, for different skeletal regions, or for subgroups of astronauts in the same mission were pooled prior to meta-analysis as follows.

#### Different measurement methods

We assumed that any method used to measure bone density provides different degrees of precision and accuracy in assessment of the same quantity. We directly assessed that bone measurements obtained in the lower limb region using projection radiography, SPA, DXA, and qCT were not significantly different (*p* = 0.57 by ANOVA) (Supplementary Fig. 1a). We excluded two studies that used ultrasound to evaluate bone density in three astronauts^24,59^ because two ultrasound measurement techniques reported inconsistent data for the same individuals. Bone formation/resorption markers measured using multiple methods at a given time point for a group of astronauts were combined as unweighted means.

#### Stratifying density measures by skeletal region

Bone density measures were grouped into four skeletal regions: skull and neck (region 1), upper limbs and thorax (region 2), lumbar vertebrae and pelvis (region 3), and lower limbs (region 4). Measurements for multiple bones in the same skeletal region for an individual or group of astronauts were pooled as unweighted means $\bar \theta _i = \frac{{\mathop {\sum }\nolimits_{j = 1}^{N_i} \theta _{i,j}}}{{N_i}}$, where *j* is the measured bone, and *N*_*i*_ is the number of bones measured in region *i*.

#### Pooling within-mission individuals and subpopulations

When outcomes were reported for multiple individuals or subgroups of astronauts for a given mission, mission-level means were obtained using sample-size weighting $\bar \theta _i = \frac{{{\sum} {(n_{i,j}\theta _{i,j})} }}{{{\sum} {n_{i,j}} }}$, where *j* is individual or subgroup within the mission *i*, and *n*_*i,j*_ is 1 for individual astronauts or the number of astronauts per subgroup. Mission-level standard deviations SD_*i*_ were computed in one of three ways:

1. Individual-level data were reported for multiple astronauts: ${\mathrm{SD}}_i = \sqrt {\frac{{\mathop {\sum }\nolimits_{j = 1}^{n_i} \left( {\bar \theta _i - \theta _{i,j}} \right)^2}}{{n_i - 1}}}$, where *θ*_*i,j*_ is the outcome for individual *j* in mission *i*, and *n*_*i*_ is the mission-level sample size.
2. Data for multiple subgroups of astronauts were reported: ${\mathrm{SD}}_i = \sqrt {\frac{{\mathop {\sum }\nolimits_{j = 1}^{N_i} \left( {\left( {n_{i,j} - 1} \right) \cdot {\mathrm{SD}}_{i,j}^2} \right)}}{{\mathop {\sum }\nolimits_{j = 1}^{N_i} \left( {n_{i,j} - 1} \right)}}}$, where SD_*i,j*_ and *n*_*i,j*_ are standard deviations and sample sizes, respectively, for subgroup *j* in mission *i*.
3. Outcome was given for a single astronaut with no variance estimate: pooled estimate of $\overline {{\mathrm{SDp}}} = \sqrt {\frac{{\mathop {\sum }\nolimits_{i = 1}^N \left( {\left( {n_i - 1} \right) \cdot {\mathrm{SD}}_i^2} \right)}}{{\mathop {\sum }\nolimits_{i = 1}^N \left( {n_i - 1} \right)}}}$, where *n*_*i*_ is the sample size for mission *i* and *N* is the number of missions.
4. For biochemical marker data, first the variation among different markers reported per individual or group of astronauts at particular time point, SD_*m*_, was computed as in step (1). Then, the variation among astronauts SD_*a*_ was computed as in step (2). The combined SD_overall_ reflected both variabilities: ${\mathrm{SD}}_{{\mathrm{overall}}} = \sqrt {{\mathrm{SD}}_m^2 + {\mathrm{SD}}_a^2}$.

### Heterogeneity and publication bias

We used $Q = \mathop {\sum }\nolimits_{i = 1}^N \left( {{\mathrm{SE}}_i^{ - 2} \cdot \left( {\bar \theta _i - \hat \theta _{{\mathrm{FE}}}} \right)^2} \right)$, where $\hat \theta _{{\mathrm{FE}}} = \frac{{\mathop {\sum }\nolimits_i {\mathrm{SE}}_i^{ - 2}\bar \theta _i}}{{\mathop {\sum }\nolimits_i {\mathrm{SE}}_i^{ - 2}}}$, $H^2 = \frac{Q}{{N - 1}}$, where *N* is number of datasets, and $I^2 = \frac{{H^2 - 1}}{{H^2}} \cdot 100\%$ to assess heterogeneity. *Q* comparison to a Chi-square distribution was used to test for homogeneity (*p*_*Q*_ ≥ 0.05). Single- and cumulative-study exclusion analysis assessed the impact of individual datasets on the overall outcome and heterogeneity, as well as homogeneity threshold (*T*_H_)^57^. Publication bias was assessed by assuming that in the absence of bias study-level outcomes have a funnel shape distribution due to random sampling error.

### Meta analysis

We used sample size weighting: $\hat \theta _N = \frac{{\mathop {\sum }\nolimits_{i = 1}^N n_i\bar \theta _i}}{{\mathop {\sum }\nolimits_{i = 1}^N n_i}}$, where $\bar \theta _i$ and *n*_*i*_ are the outcomes and sample sizes for mission *i*, *N* is the number of datasets. Standard deviation was $\widehat {{\mathrm{SD}}}_N = \frac{{\mathop {\sum }\nolimits_{i = 1}^N \left( {\left( {n_{i,} - 1} \right) \cdot {\mathrm{SD}}_i^2} \right)}}{{\mathop {\sum }\nolimits_{i = 1}^N \left( {n_i - 1} \right)}}$; standard error: $\widehat {{\mathrm{SE}}}_N = \frac{{\widehat {{\mathrm{SD}}}_N}}{{\sqrt N }}$; and 95% confidence intervals (CI) $= \pm z_{(1 - \alpha /2)} \cdot \widehat {{\mathrm{SE}}}_N = \pm 1.96 \cdot \widehat {{\mathrm{SE}}}_N$.

### Subgroup analysis

When specified, outcomes were grouped into *k* bins, and binned means $\hat \theta _k = \frac{{{\sum} {n_{k,i}\bar \theta _{k,i}} }}{{{\sum} {n_{k,i}} }}$ and standard deviations $\widehat {{\mathrm{SD}}}_k = \frac{{{\sum} {\left( {\left( {n_{k,i} - 1} \right) \cdot {\mathrm{SD}}_{k,i}^2} \right)} }}{{{\sum} {\left( {n_{k,i} - 1} \right)} }}$ were computed, where $\bar \theta _{k,i}$, *n*_*k,i*_, and SD_*k,i*_ were the outcome, sample size, and standard deviation reported for study *i* belonging to bin *k*. The division for the subgroup analysis was performed to achieve approximately equal size group in each category.

### Meta-regression and Monte-Carlo model fitting

Between-study meta-regression was performed assuming a random effects model: *y*_*i*_ = *β*_0_ + *β*_1_*x*_*i*_ + *ε*_*j*_ + *η*_*j*_, where *β*_0_ was fixed at 0 (0% from pre-flight on day 0 of spaceflight), *β*_1_ describes the relationship between *x*_*i*_ (mission duration) and outcome *y*_*i*_; *ε*_*j*_ and *η*_*j*_ are intra- and inter-study variabilities approximated by ${\cal{N}}(0,{\mathrm{SE}}_i^2)$, and ${\cal{N}}(0,\tau ^2)$, *τ*^2^ was computed using DerSimonian and Laird estimator^60^. For fitting a non-linear model, or considering additional variance for a linear relationship, a Monte-Carlo error propagation method^61^ was used with MetaLab^57^, or a custom MATLAB script for piecewise functions (Supplementary Methods 3). For in-flight changes in resorption markers sigmoidal function was used $y = \frac{{\beta _1\left( t \right)^{\beta _3}}}{{\left( {\beta _2} \right)^{\beta _3} + \left( t \right)^{\beta _3}}}$, where *β*_1_ is the maximum in-flight change, *β*_2_ is time to half-maximal change, and *β*_3_ defines the steepness. For post-flight change in resorption markers we used exponential function $y = \beta _0e^{\beta _1t}$, where *β*_0_ was the last in-flight data point, and *β*_1_ a decay constant. Changes in formation markers, and agreement between markers was modeled using linear function *y* = *β*_0_ + *β*_1_*x*, where *β*_0_ was the last in-flight data point for post-flight changes in formation markers.

### Outcome reporting and sample size calculations

Data are presented as means with lower and upper limits of 95% CI as: mean [lower CI, upper CI]. Outcome variability was assessed using coefficient of variance ${\mathrm{CV}} = 100 \times \widehat {{\mathrm{SD}}_N}/\left| {\hat \theta _N} \right|$. Using meta-analytic outcomes, sample sizes required to detect changes with 80% power (*β* = 0.80) and 95% significance level (*α* = 0.05) were calculated using the samplesizepwr function in MATLAB.

## Supplementary information

reporting-summary

Supplementary information

## Data Availability

Raw data can be made available to a reader upon reasonable request.

## Code availability

Custom MATLAB code used to fit piece-wise functions to biochemical bone resorption and formation data post-flight can be found in Supplementary Methods 3.
