# Supplementary material for: A systematic review and meta-analysis of bone loss in space travelers
Source: NPJ Microgravity. 2020 May 5;6:13. doi: 10.1038/s41526-020-0103-2 (PMC7200725; doi:10.1038/s41526-020-0103-2)
Supplement: Supplementary file 2 — Supplementary information [file 41526_2020_103_MOESM2_ESM.pdf]

Supplementary material for the manuscript *"A systematic review and meta-analysis of bone loss in space travelers"* by Mariya Stavnichuk, Nicholas Mikolajewicz, Tatsuya Corlett, Martin Morris, Svetlana V. Komarova

Supplementary Figures

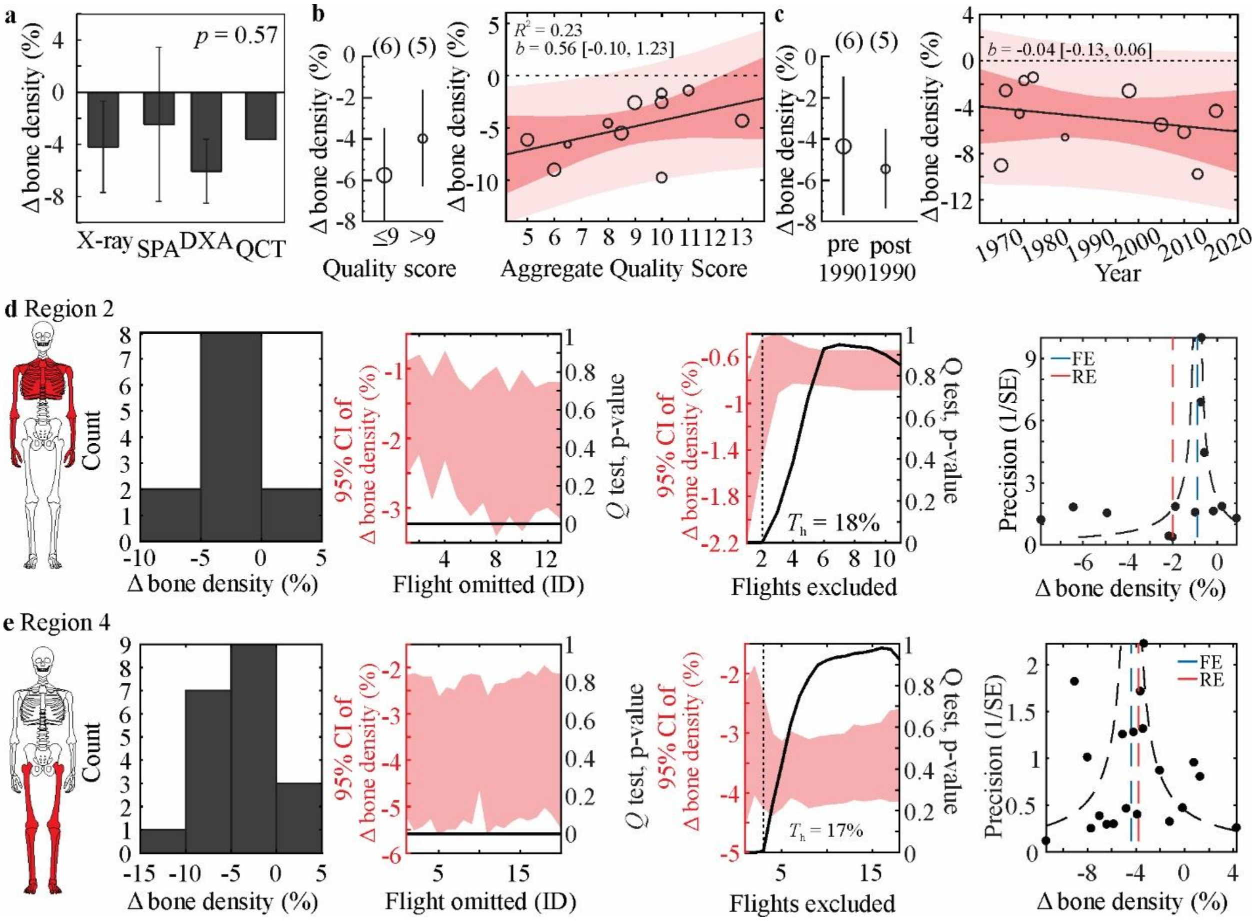

Supplementary Figure 1. Quality analysis of bone density data. (a) A comparison of bone density in the lower limb region obtained using different imaging radiography techniques, *p*-value was generated by single-factor ANOVA. **(b, c)** Effect of study quality **(b)** and publication year **(c)** on estimates of spaceflight related bone loss in lower limbs (% pre-flight) was assessed by subgroup analysis (*left*) and meta-regression (*right*). For subgroup analyses shown are subgroup estimates ± 95% CI, (n) is a number of studies in a subgroup. For meta-regression, *circles* are study-level estimates, *dark/light red bands*: 95% confidence/prediction intervals; *β*: meta-regression slope. Marker sizes are proportional to the number of astronauts. **(d-e)** For region 2 (upper body **(d)**), and region 4 (lower limbs **(e)**), study-level outcome distributions (*first column*), effect of single-/cumulative-study exclusion on random effects (RE) estimates and heterogeneity of outcomes (*second and third columns, respectively*), and funnel plots for study-level outcomes

(*fourth column*) are shown. For single/cumulative exclusion plots, *red band*: 95% CI for studies remaining after exclusion of a single study or cumulative exclusion of the most heterogeneous studies according to maximal *Q*-reduction criteria, *black curve*: *p*-value for *Q*-heterogeneity test, *dashed black line*: homogeneity threshold T_H. For funnel plots, *black markers*: study-level data, *blue lines*: fixed effect (FE) estimate, *red lines*: RE estimate, *black lines*: theoretical 95% CI for FE estimate in the absence of bias. Dataset used to generate this figure does not contain data from one study included in the analysis after the search update.

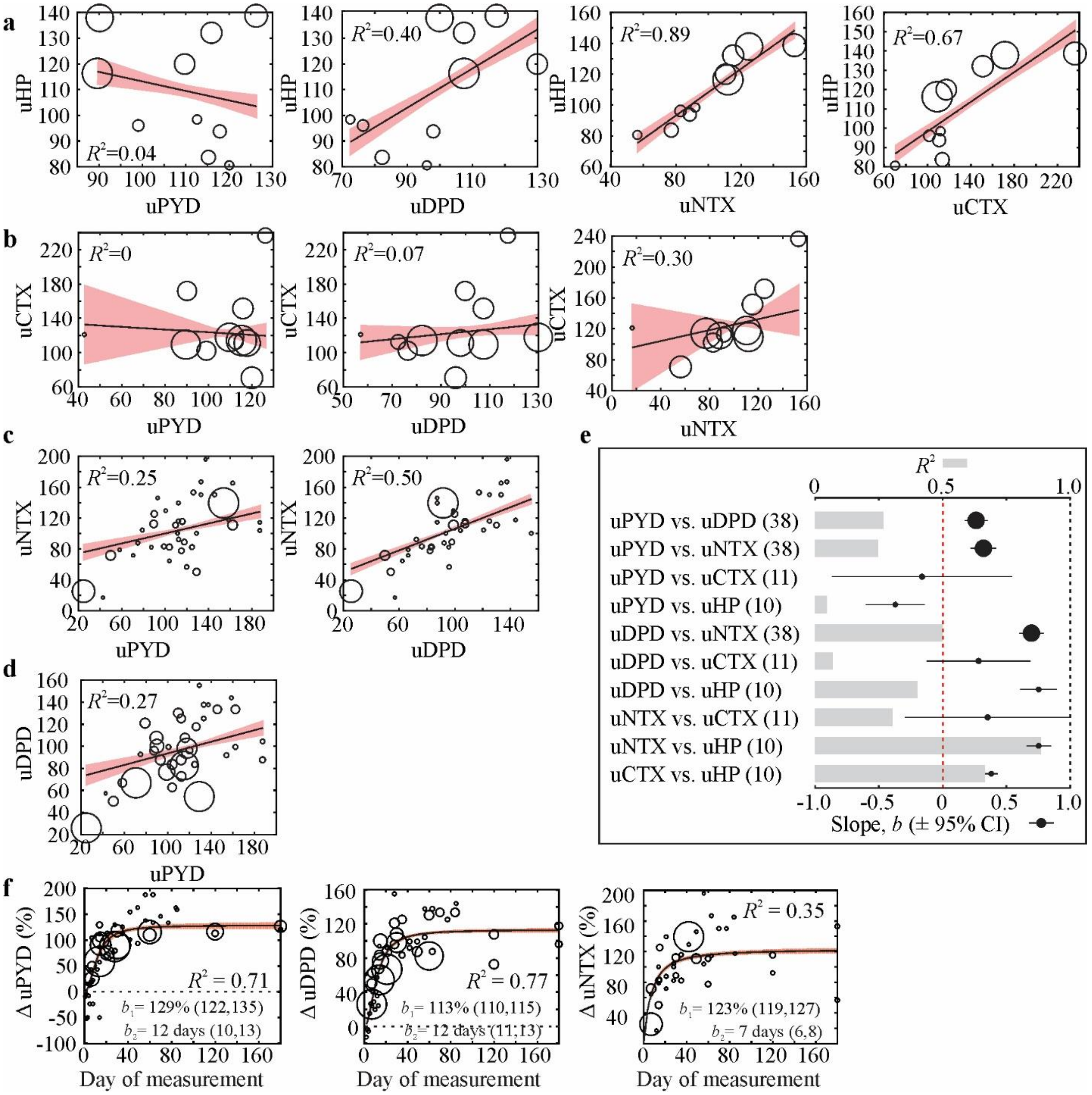

Supplementary Figure 2. Correlation and agreement between different markers of resorption. (a-d) Correlations between markers uHP, uCTX, uNTX, uDPD and uPYD were examined. *Black markers*: mission-level changes, marker sizes are proportional to weights, *black line/red bands*: linear regression/95% CI, R^2: regression coefficient. **(e)** Forest plot of regression

slopes β ± 95% CI (*bottom axes*) overlaid with horizontal bar plot showing corresponding regression coefficients R^2 (*top axes*) for each pair of markers. Marker sizes are proportional to sample size (shown in parentheses). *Red dashed line*: β = 0 reference, *black dashed line*: β = 1 reference. **(f)** Relationship between in-flight day of measurement and changes in uPYD, uDPD, uNTX markers (% pre-flight) fit to a sigmoidal function using the Monte Carlo method. Marker sizes proportional to the number of astronauts, *black line/red band*: mean fit/95% CI, β_1: estimate of biochemical marker maximum level, β_2: estimate of the time to half-maximum. Dataset used to generate this figure does not contain data from one study included in the analysis after the search update.

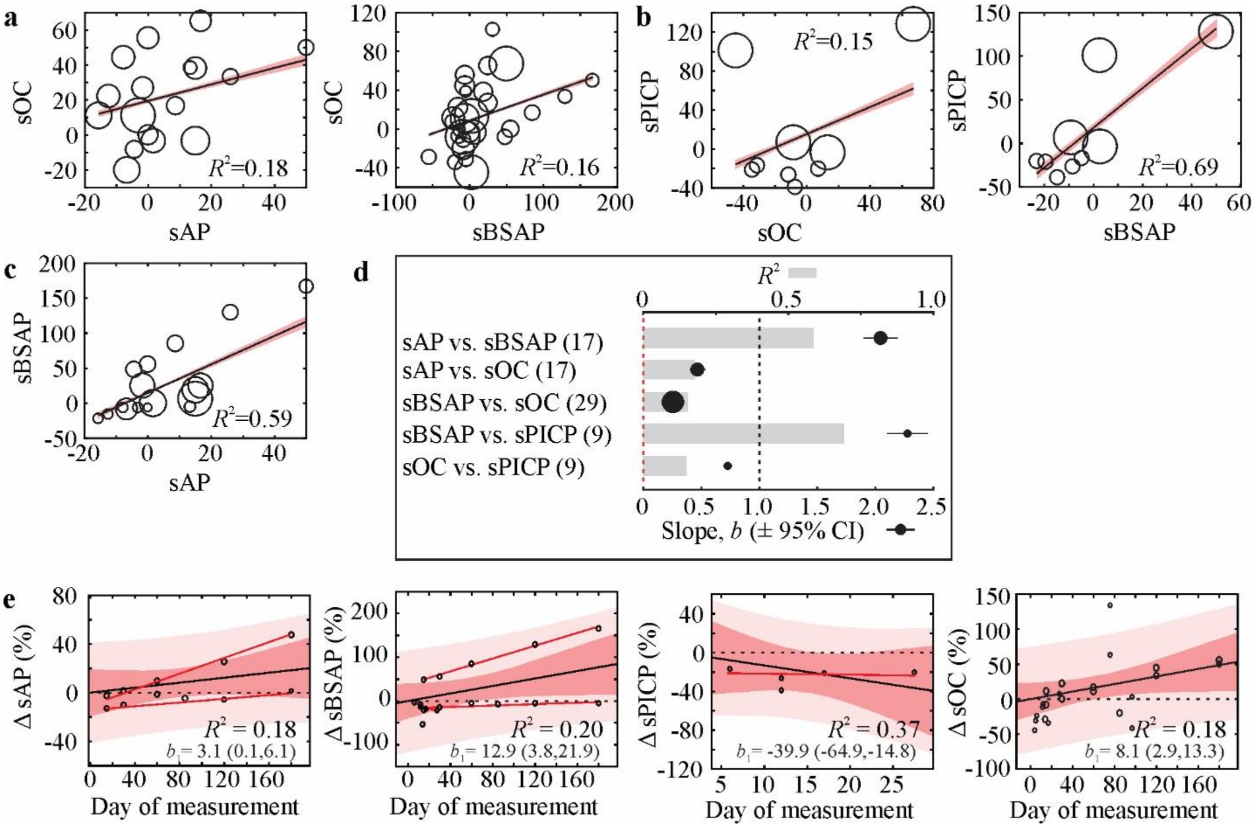

Supplementary Figure 3. Correlation and agreement between different markers of formation. (a-c) Correlation between markers sAP, sBSAP, sOC and sPICP was examined. *Black markers*: mission-level data, marker sizes are proportional to weights, *black line/red bands*: linear regression/95% CI, R^2*:* regression coefficient. **(d)** Forest plot of regression slopes β ± 95% CI (*bottom axes*) overlaid with horizontal bar plot showing corresponding regression coefficients R^2 (*top axes*) for each pair of markers. Marker sizes are proportional to sample size (shown in parentheses). *Red dashed vertical line*: β = 0 reference, *black dashed vertical line*: β = 1 reference. **(e)** Relationship between in-flight day of measurement and changes in sAP, sBSAP, sPICP, and sOC markers (% pre-flight) assessed by meta-regression. Marker sizes proportional to the number of astronauts, *black line*: meta-regression forced through a zero intercept, *dark/light red bands*: 95% confidence/prediction intervals, *red lines*: within-study regressions, β_1: the rate of change ± 95% CI estimated between-studies. Dataset used to generate this figure does not contain data from one study included in the analysis after the search update.

Supplementary Tables

Supplementary Table 1. Spaceflight-related bone density changes in different skeletal regions.

Study	Mission	Duration (days)	n	Δ bone density (% ± 95% CI)
Region 1: Skull & Cervical Spine				
Oganov 2005	MIR EO-6-24	180	32	2.2 (1.1, 3.3)
Region 2: Upper Limb & Thorax				
Mack 1971	Gemini 7	14	2	-6.4 (-7.5, -5.4)
Mack 1971	Apollo 7	11	3	-2.2 (-6.9, 2.6)
Mack 1971	Apollo 8	6	3	-7.9 (-9.5, -6.2)
Biryukov 1970	Souyz 9	18	2	-4.9 (-6.2, -3.6)
Vogel 1975	Apollo 14	9	3	0.2 (-0.8, 1.3)
Vogel 1975	Apollo 15	12	3	-1.9 (-2.9, -0.8)
Vogel 1975	Apollo 16	11	3	-0.7 (-0.9, -0.5)
Johnston 1977	Skylab 2	28	3	0.9 (-0.7, 2.4)
Johnston 1977	Skylab 3	59	3	-0.7 (-1.0, -0.4)
Johnston 1977	Skylab 4	84	3	-0.2 (-1.4, 1.0)
Miyamoto 1998	STS 72	8	1	-2.0 (-7.3, 3.3)
Oganov 2005	MIR EO-6-24	180	31	-1.0 (-2.2, 0.3)
Vico 2017	ISS 15-35	252	13	-0.6 (-1.0, -0.1)
**Overall**	**I2 =95%, H2 = 21, p_Q<0.001**		**73**	**-1.4 (-2.1, -0.6)**
**>28 days**	**I2 =63%, H2 = 3, p_Q=0.028**		**53**	**-0.7 (-1.3, -0.2)**
Region 3: Pelvis & Lumbar Spine				
Oganov 2005	Soyuz T7	211	3	-4.1 (-7.9, -0.4)
Oganov 2005	Soyuz T9	150	2	-6.6 (-14.9, 1.8)
Oganov 2005	Soyuz T11	237	2	-4.5 (-9.4, 0.4)
Miyamoto 1998	STS 65	237	2	-2.5 (-11.7, 6.7)
Miyamoto 1998	STS 72	237	2	-3.5 (-12.7, 5.7)
Oganov 2005	MIR EO-6-24	180	28	-5.9 (-7.3, -4.5)
LeBlanc 2013	ISS 1-8	150	18	-9.8 (-12.5, -7.1)
Vico 2017	ISS 15-35	252	13	-5.1 (-7.6, -2.5)
Sibonga 2019	ISS 30-49	n/d	10	-3.3 (-4.7, -2.0)
**Overall**	**I2 =98%, H2 = 56, p_Q<0.001**		**78**	**-6.2 (-6.7, -5.6)**
Region 4: Lower Limbs				
Vose 1974	Gemini 4	4	2	-3.4 (-4.2, -2.5)
Vose 1974	Gemini 5	8	2	-5.9 (-12.4, 0.7)
Mack 1971	Gemini 7	14	2	-4.2 (-5.7, -2.7)
Mack 1971	Apollo 7	11	3	-0.2 (-4.3, 4.0)
Vogel 1975	Soyuz 3	3	1	-7.7 (-15.5, 0.1)
Mack 1971	Apollo 8	6	3	-3.4 (-4.9, -1.9)
Biryukov 1970	Soyuz 9	18	2	-9.1 (-10.1, -8.0)
Vogel 1975	Apollo 14	9	3	1.3 (-1.2, 3.7)
Vogel 1975	Apollo 15	12	3	-4.8 (-9.0, -0.6)
Vogel 1975	Apollo 16	11	3	4.3 (-3.3, 11.8)

Johnston 1977	Skylab 2	28	3	0.8 (-1.3, 2.8)
Johnston 1977	Skylab 3	59	3	-1.2 (-7.3, 4.8)
Johnston 1977	Skylab 4	84	3	-3.9 (-8.8, 1.0)
Stupakov 1984	Soyuz 31	140	2	-11.4 (-27.9, 5.1)
Stupakov 1984	Soyuz 34	175	2	-6.4 (-13.1, 0.3)
Stupakov 1984	Soyuz 37	184	2	-7.0 (-12.1, -1.9)
Stupakov 1984	Soyuz T-4	75	2	-2.1 (-4.3, 0.2)
Oganov 2005	MIR EO-6-24	180	31	-8.0 (-9.9, -6.1)
Ellman 2010	ISS 1-18	171	25	-5.1 (-6.7, -3.5)
Vico 2017	ISS 15-35	252	13	-3.6 (-4.8, -2.5)
Sibonga 2019	ISS 30-49	n/d	10	-3.3 (-3.9, -2.6)
**Overall**	**I2 =98%, H2 = 56, p_Q<0.001**		**120**	**-4.9 (-5.6, -4.2)**
**>28 days**	**I2 =98%, H2 = 58, p_Q<0.001**		**96**	**-5.4 (-6.0, -4.9)**

Supplementary Table 2. Spaceflight-related bone density changes in different bones.

**Bone**	**Study**	**Mission**	**Duration (days)**	**n**	**Δ bone density (% ± 95% CI)**
Region 2: Upper Limb & Thorax					
**Ulna**	Mack 1971	Apollo 7	11	3	-1.4 (-4.9, 2.0)
	Mack 1971	Apollo 8	6	3	-11.7 (-17.3, -6.1)
	Vogel 1975	Apollo 14	9	3	-0.5(-1.6, 0.6)
	Vogel 1975	Apollo 15	12	3	-2.3 (-3.6, -0.9)
	Vogel 1975	Apollo 16	11	3	-3.0 (-3.8, -2.2)
	Johnston 1977	Skylab 2	28	3	1.4 (-1.0, 3.7)
	Johnston 1977	Skylab 3	59	3	-0.5 (-1.7, 0.6)
	Johnston 1977	Skylab 4	84	3	-0.1 (-1.9, 1.7)
Overall	I2 = 98%, H2 =49, p_Q<0.001			24	-2.3 (-3.4, -1.1)
**Radius**	Mack 1971	Apollo 7	11	3	-1.2 (-5.6, 3.2)
	Mack 1971	Apollo 8	6	3	-10.4 (-12.0, -8.8)
	Vogel 1975	Apollo 14	9	3	-0.5 (-1.6, 0.6)
	Vogel 1975	Apollo 15	12	3	-2.3 (-3.6, -0.9)
	Vogel 1975	Apollo 16	11	3	-3.0 (-3.8, -2.2)
	Johnston 1977	Skylab 2	28	3	0.4 (-0.7, 1.5)
	Johnston 1977	Skylab 3	59	3	-0.9 (-2.0, 0.2)
	Johnston 1977	Skylab 4	84	3	-0.2 (-1.5, 1.0)
	Miyamoto 1998	STS 72	8	1	-2.0 (-11.2, 7.2)
	Vico 2017	ISS 15-35	252	13	-0.6 (-1.0, -0.1)
Overall	I2 = 97%, H2 = 34, p_Q<0.001			38	-1.1 (-2.1, -0.2)
**Capitate**	Mack 1971	Gemini 7	14	2	-6.8 (-11.7, -1.9)
	Mack 1971	Apollo 7	11	3	-1.4 (-6.0, 3.2)
	Mack 1971	Apollo 8	6	3	-9.5 (-12.5, -6.4)
Overall	I2 = 98%, H2 = 50, p_Q<0.001			8	-5.8 (-7.0, -4.6)

**Phalanx**	Mack 1971	Gemini 7	14	2	-6.2 (-7.1, -5.4)
	Mack 1971	Apollo 7	11	3	-4.6 (-11.3, 2.1)
	Mack 1971	Apollo 8	6	3	0.1 (-4.6, 4.7)
	Biryukov 1970	Souyz 9	18	2	-4.9 (-6.2, -3.6)
Overall	I2 = 95%, H2 = 20, p_Q<0.001			10	-3.6 (-4.8, -2.4)
**Thoracic vertebrae**	Oganov 2005	MIR EO-6-24	180	31	-1.0 (-2.2, 0.3)
Region 3: Pelvis & Lumbar Spine					
**Lumbar vertebrae**	Oganov 2005	Soyuz T7	211	3	-4.1 (-7.9, -0.4)
	Oganov 2005	Soyuz T9	150	2	-6.6 (-14.9, 1.8)
	Oganov 2005	Soyuz T11	237	2	-4.5 (-9.4, 0.4)
	Miyamoto 1998	STS 65	237	2	-2.5 (-11.7, 6.7)
	Miyamoto 1998	STS 72	237	2	-3.5 (-12.7, 5.7)
	Oganov 2005	MIR EO-6-24	180	28	-5.9 (-7.3, -4.5)
	Vico 2017	ISS 15-35	252	13	-4.6 (-7.9, -1.3)
	Sibonga 2019	ISS 30-49	n/d	10	-2.9 (-3.3, -2.4)
Overall	I2 = 92%, H2 = 12, p_Q<0.001			68	-4.9 (-5.5, -4.3)
**Hip bone**	LeBlanc 2013	ISS 1-8	150	18	-9.8 (-12.5, -7.1)
	Vico 2017	ISS 15-35	252	13	-5.5 (-7.0, -4.0)
	Sibonga 2019	ISS 30-49	n/d	10	-3.8 (-4.2, -3.4)
Overall	I2 = 99%, H2 = 136, p_Q<0.001			31	-7.0 (-7.5, -6.5)
Region 4: Lower Limbs					
**Femur**	Oganov 2005	MIR EO-6-24	180	31	-8.0 (-9.9, -6.1)
	Ellman 2010	ISS 1-18 ♂	171	20	-6.9 (-11.7, -2.1)
	Ellman 2010	ISS 1-18 ♀	171	5	-3.3 (-7.1, 0.5)
	Vico 2017	ISS 15-35	252	13	-4.7 (-5.1, -4.4)
	Sibonga 2019	ISS 30-49	n/d	10	-3.3 (-3.9, -2.6)
Overall	I2 = 98%, H2 = 66, p_Q<0.001			69	-6.3 (-6.8, -5.8)
**Tibia**	Vico 2017	ISS 15-35	252	13	-2.5 (-4.1, -1.0)
**Talus**	Mack 1971	Gemini 7	14	2	-5.5 (-8.5, -2.5)
	Mack 1971	Apollo 7	11	3	0.4 (-3.6, 4.3)
	Mack 1971	Apollo 8	6	3	-2.9 (-3.2, -2.5)
Overall	I2 =95%, H2 = 22, p_Q<0.001			8	-2.3 (-3.5, -1.1)
**Calcaneus**	Vose 1974	Gemini 4	4	2	-3.4 (-4.2, -2.5)
	Vose 1974	Gemini 5	8	2	-5.9 (-12.4, 0.7)

	Mack 1971	Gemini 7	14	2	-2.9 (-2.9, -2.8)
	Mack 1971	Apollo 7	11	3	-0.7 (-5.1, 3.7)
	Vogel 1975	Soyuz 3	3	1	-7.7 (-16.9, 1.5)
	Mack 1971	Apollo 8	6	3	-4.0 (-7.0, -1.0)
	Biryukov 1970	Soyuz 9	18	2	-9.1 (-10.1, -8.0)
	Vogel 1975	Apollo 14	9	3	1.3 (-1.2, 3.7)
	Vogel 1975	Apollo 15	12	3	-4.8 (-9.0, -0.6)
	Vogel 1975	Apollo 16	11	3	4.3 (-3.3, 11.8)
	Johnston 1977	Skylab 2	28	3	0.8 (-1.3, 2.8)
	Johnston 1977	Skylab 3	59	3	-1.2 (-7.3, 4.8)
	Johnston 1977	Skylab 4	84	3	-3.9 (-8.8, 1.0)
	Stupakov 1984	Soyuz 31	140	2	-11.4 (-27.9, 5.1)
	Stupakov 1984	Soyuz 34	175	2	-6.4 (-13.1, 0.3)
	Stupakov 1984	Soyuz 37	184	2	-7.0 (-12.1, -1.9)
	Stupakov 1984	Soyuz T-4	75	2	-2.1 (-4.3, 0.2)
Overall	I2 = 97%, H2 = 38, p_Q<0.001			41	-3.1 (-4.4, -1.9)

Supplementary Methods

1. Search strategy

1. ((andre-deshays adj3 (claudie or haignere)) or (acaba adj3 joseph) or (acton adj3 (loren or wilbur)) or (adamson adj3 james) or (afanasyev adj3 (viktor or mikhaylovich)) or (a?dyn adj3 aimbetov) or (akers adj3 thomas) or (akiyama adj3 toyohiro) or (aksyonov adj3 vladimir) or (al-saud adj3 (sultan or salman)) or (aldrin adj3 (buzz or edwin or eugene)) or (aleksandr adj3 (panayotovi or aleksandrov)) or (aleksandrov adj3 aleksandr) or (aleksey adj3 ovchinin) or (allen adj3 andrew) or (allen adj3 joseph) or (altman adj3 scott) or (anders adj3 (william or alison)) or (anderson adj3 clayton) or (anderson adj3 michael) or (ansari adj3 anousheh) or (antonelli adj3 dominic) or (apt adj3 jerome) or (archambault adj3 lee) or (armstrong adj3 neil) or (arnaldo adj3 (tamayo or mendez)) or (arnold adj3 richard) or (artemyev adj3 oleg) or (artsebarsky adj3 anatoly) or (artyukhin adj3 yuri) or (ashby adj3 jeffrey) or (atkov adj3 oleg) or (aubakirov adj3 toktar) or (avdeyev adj3 sergei)).ti,ab,kw.

2. ((bagian adj3 james) or (baker adj3 (ellen or michael)) or (balandin adj3 aleksandr) or (barratt adj3 michael) or (barry adj3 daniel) or (bartoe adj3 (john-david or francis)) or (baturin adj3 yuri) or (baudry adj3 patrick) or (bean adj3 (alan or lavern)) or (behnken adj3 robert) or (bella adj3 ivan) or (belyayev adj3 pavel) or (beregovoi adj3 georgi) or (berezovoy adj3 anatoly) or (bertalan adj3 farkas) or (bjarni adj3 tryggvason) or (blaha adj3 john) or (bloomfield adj3 michael) or (bluford adj3 guion) or (bobko adj3 karol) or (boe adj3 eric) or (bolden adj3 charles) or (bondar adj3 (roberta or lynn)) or (borisenko adj3 andrei) or (borman adj3 frank) or (bowen adj3 stephen) or (bowersox adj3 kenneth) or (brady adj3 charles) or (brand adj3 vance) or (brandenstein adj3 daniel) or (bresnik adj3 randolph) or (bridges adj3 roy) or (brown adj3 (curtis or david or mark)) or (buchli adj3 james) or (buckey adj3 (jay or clark)) or (budarin adj3 nikolai) or (burbank adj3 daniel) or (bursch adj3 daniel) or (bykovsky adj3 valery)).ti,ab,kw.

3. ((cabana adj3 robert) or (caldwell adj3 tracy) or (camarda adj3 charles) or (cameron adj3 kenneth) or (carey adj3 duane) or (carpenter adj3 scott) or (carr adj3 gerald) or (carter adj3 (manley or lanier)) or (casper adj3 john) or (cassidy adj3 chris*) or (cenker adj3 robert) or (cernan adj3 eugene) or (chamitoff adj3 gregory) or (chang-diaz adj3 franklin) or (chawla adj3 kalpana) or (cheli adj3 maurizio) or (chiao adj3 leroy) or (chilton adj3 kevin) or (chris adj3 hadfield) or (christer adj3 fuglesang) or (chretien adj3 jean-loup) or (clark adj3 laurel) or (cleave adj3 mary) or (clervoy adj3 jean-francois) or (clifford adj3 michael) or (coats adj3 michael) or (cockrell adj3 kenneth) or (coleman adj3 catherine) or (collins adj3 (eileen or michael)) or (conrad adj3 charles) or (cooper adj3 gordon) or (covey adj3 richard) or (creamer adj3 timothy) or (creighton adj3 john) or (crippen adj3 robert) or (cristofretti adj3 samantha) or (crouch adj3 (roger or keith)) or (culbertson adj3 frank) or (cunningham adj3 (ronnie or walter)) or (curbeam adj3 robert) or (currie adj3 nancy)).ti,ab,kw.

4. ((davis adj3 (nancy or jan)) or ("de winne" adj3 frank) or (delucas adj3 (lawrence or james)) or (dezhurov adj3 vladimir) or (dobrovolski adj3 georgi) or (doi adj3 takao) or (drew adj3 (alvin or benjamin)) or (duffy adj3 brian) or (duke adj3 (charles or moss)) or (dumitru adj3 prunariu) or (dunbar adj3 bonnie) or (duque adj3 pedro) or (durrance adj3 (samuel or thornton)) or (dutton adj3 james) or (dyomin adj3 lev) or (dzhanibekov adj3 vladimir) or (edwards adj3 joe) or (eisele adj3

donn) or (england adj3 anthony) or (engle adj3 joe) or (evans adj3 ronald) or (ewald adj3 reinhold) or (eyharts adj3 leopold) or (fabian adj3 john) or (faris adj3 achmed) or (favier adj3 jean-jacques) or (fei adj3 junlong) or (feoktistov adj3 konstantin) or (ferguson adj3 christopher) or (fettman adj3 (martin or joseph)) or (feustel adj3 andrew) or (filipchenko adj3 anatoly) or (fincke adj3 michael) or (fisher adj3 (anna or william)) or (flade adj3 klaus-dietrich) or (foale adj3 michael) or (ford adj3 kevin) or (foreman adj3 michael) or (forrester adj3 patrick) or (fossum adj3 michael) or (franco adj3 malerba) or (frick adj3 stephen) or (frimout adj3 (dirk or dries or david)) or (fullerton adj3 charles) or (furrer adj3 (reinhard or alfred)) or (furukawa adj3 satoshi)).ti,ab,kw.

5. ((gaffney adj3 andrew) or (gagarin adj3 yuri) or (garan adj3 ronald) or (gardner adj3 (dale or guy)) or (garn adj3 (edwin or jacob)) or (garneau adj3 (marc or joseph or jean-pierre)) or (garriott adj3 owen) or (garriott adj3 (richard or allen)) or (gemar adj3 charles) or (georgi adj3 ivanov) or (gernhardt adj3 michael) or (gerst adj3 alexander) or (gibson adj3 edward) or (gibson adj3 robert) or (gidzenko adj3 yuri) or (glazkov adj3 yuri) or (glenn adj3 john) or (godwin adj3 linda) or (good adj3 michael) or (gorbatko adj3 viktor) or (gordon adj3 richard) or (gorie adj3 dominic) or (grabe adj3 ronald) or (grechko adj3 georgi) or (gregory adj3 frederick) or (gregory adj3 william) or (griggs adj3 david) or (grissom adj3 virgil) or (grunsfeld adj3 john) or (gubarev adj3 aleksei) or (guidoni adj3 umberto) or (gutierrez adj3 sidney) or (guy adj3 laliberte) or (haignere adj3 jean-pierre) or (haise adj3 fred) or (halsell adj3 james) or (ham adj3 kenneth) or (hammond adj3 blaine) or (harbaugh adj3 gregory) or (harris adj3 bernard) or (hart adj3 terry) or (hartsfield adj3 henry) or (hauck adj3 fred*) or (hawley adj3 steven) or (helen adj3 sharman) or (helms adj3 susan) or (henize adj3 karl) or (hennen adj3 (thomas or john)) or (henricks adj3 terence) or (hernandez adj3 jose) or (herrington adj3 john) or (hieb adj3 richard) or (higginbotham adj3 joan) or (hilmers adj3 david) or (hire adj3 kathryn) or (hobaugh adj3 charles) or (hoffman adj3 jeffrey) or (hopkins adj3 (michael or scott)) or (horowitz adj3 scott) or (hoshide adj3 akihiko) or (hughes-fulford adj3 (millie or elizabeth)) or (hurley adj3 douglas) or (husband adj3 rick)).ti,ab,kw.

6. ((irwin adj3 james) or (ivanchenkov adj3 aleksandr) or (ivanishin adj3 anatoli) or (ivins adj3 marsha) or (jahn adj3 sigmund) or (jarvis adj3 (gregory or bruce)) or (jemison adj3 mae) or (jernigan adj3 tamara) or (jett adj3 brent) or (jing adj3 haipeng) or (johnson adj3 gregory) or (jones adj3 thomas) or (jugderdemidiin or gurragchaa) or (kadenyuk adj3 leonid) or (kaleri adj3 aleksandr) or (kavandi adj3 janet) or (kelly adj3 james) or (kelly adj3 mark) or (kelly adj3 scott) or (kerwin adj3 joseph) or (khrunov adj3 yevgeny) or (kilrain-still adj3 susan) or (kimbrough adj3 robert) or (kizim adj3 leonid) or (klimuk adj3 pyotr) or (komarov adj3 vladimir) or (kondakova adj3 yelena) or (kondratyev adj3 dmitri) or (kononenko adj3 oleg) or (kopra adj3 timothy) or (korniyenko adj3 mikhail) or (korzun adj3 valery) or (kotov adj3 oleg) or (kovalyonok adj3 vladimir) or (kozeyev adj3 konstantin) or (kregel adj3 kevin) or (krikalev adj3 sergei) or (kubasov adj3 valeri) or (kuipers adj3 andre) or (laveykin adj3 aleksandr) or (lawrence adj3 wendy) or (lazarev adj3 vasili) or (lazutkin adj3 aleksandr) or (lebedev adj3 valentin) or (lee adj3 mark) or (leestma adj3 david) or (lenoir adj3 william) or (leonov adj3 aleksei) or (leslie adj3 (fred or weldon)) or (levchenko adj3 anatoli) or (lichtenberg adj3 (byron or kurt)) or (lind adj3 don) or (lindgren adj3 (kjell or norwood)) or (lindsey adj3 steven) or (linenger adj3 jerry) or (linnehan adj3 richard) or (linteris adj3 (gregory or thomas)) or (liu adj3 (boming or wang or yang)) or (lockhart adj3 paul) or (lodewijk adj3 "van der berg") or (lonchakov adj3 yuri) or (lopez-alegria adj3

michael) or (lounge adj3 john) or (lousma adj3 jack) or (love adj3 stanley) or (lovell adj3 james) or (low adj3 david) or (lu adj3 edward) or (lucid adj3 shannon) or (lyakhov adj3 vladimir)).ti,ab,kw.

7. ((magnus adj3 sandra) or (makarov adj3 oleg) or (malenchenko adj3 yuri) or (malyshev adj3 yuri) or (manakov adj3 gennadi) or (manarov adj3 musa) or (marshburn adj3 thomas) or (massimino adj3 michael) or (mastracchio adj3 richard) or (mattingly adj3 thomas) or (mcarthur adj3 megan) or (mcarthur adj3 william) or (mcauliffe adj3 (sharon or christa or corrigan)) or (mcbride adj3 jon) or (mccandless adj3 bruce) or (mccool adj3 william) or (mcculley adj3 michael) or (mcdivitt adj3 james) or (mcmonagle adj3 donald) or (mcnair adj3 ronald) or (meade adj3 carl) or (melnick adj3 bruce) or (melroy adj3 pamela) or (melvin adj3 leland) or (merbold adj3 ulf) or (messerschmid adj3 (ernst or willi)) or (metcalf-lindenburger adj3 dorothy) or (miroslaw adj3 hermaszewske) or (misurikin adj3 aleksandr) or (mitchell adj3 edgar) or (mogensen adj3 andreas) or (mohmand adj3 (abdul or ahad)) or (mohri adj3 mamoru) or (morgan adj3 barbara) or (morin adj3 lee) or (morukov adj3 boris) or (mukai adj3 chiaki) or (mullane adj3 richard) or (musabayev adj3 talgat) or (musgrave adj3 (franklin or story)) or (nagel adj3 steven) or (nelson adj3 george) or (nelson adj3 (william or clarence)) or ("neri vela" adj3 rodolfo) or (nespoli adj3 paolo) or (newman adj3 james) or (nicollier adj3 claude) or (nie adj3 haisheng) or (nikolayev adj3 andriyan) or (noguchi adj3 soichi) or (noriega adj3 carlos) or (novitski adj3 oleg) or (nowak adj3 lisa) or (nyberg adj3 karen) or (o'connor adj3 bryan) or (ochoa adj3 ellen) or (ockels adj3 (wubbo or johannes)) or (oefelein adj3 william) or (olivas adj3 john) or (olsen adj3 (gregory or hammond)) or (onishi adj3 takuya) or (onizuka adj3 ellison) or (onufrienko adj3 yuri) or (oswald adj3 stephen) or (overmyer adj3 robert) or (padalka adj3 gennady)).ti,ab,kw.

8. ((pailes adj3 (william or arthur)) or (parazynski adj3 scott) or (parise adj3 (ronald or anthony)) or (parker adj3 robert) or (parmitano adj3 luca) or (patrick adj3 nicholas) or (patsayev adj3 viktor) or (pawelczyk adj3 (james or anthony)) or (payette adj3 julie) or (payton adj3 (gary or eugene)) or (peake adj3 (timothy or nigel)) or (perrin adj3 philippe) or (peterson adj3 donald) or (pettit adj3 donald) or (pham adj3 tuan) or (phillips adj3 john) or (pogue adj3 william) or (poindexter adj3 alan) or (polansky adj3 mark) or (poleshchuk adj3 aleksandr) or (polyakov adj3 valeri) or (pontes adj3 (marcos or cesar)) or (popov adj3 leonid) or (popovich adj3 pavel) or (precourt adj3 charles) or (rakesh adj3 sharma) or (ramon adj3 ilan) or (readdy adj3 william) or (reightler adj3 kenneth) or (reilly adj3 james) or (reisman adj3 garrett) or (reiter adj3 thomas) or (resnik adj3 judith) or (revin adj3 sergei) or (richards adj3 paul) or (richards adj3 richard) or (ride adj3 sally) or (robert adj3 thirsk) or (robinson adj3 stephen) or (romanenko adj3 roman) or (romanenko adj3 yuri) or (rominger adj3 kent) or (roosa adj3 stuart) or (ross adj3 jerry) or (rozhdestvensky adj3 valery) or (rubins adj3 (kathleen or hallisey)) or (rukavishnikov adj3 nikolai) or (runco adj3 mario) or (ryazanski adj3 sergei) or (ryumin adj3 valery)).ti,ab,kw.

9. ((sacco adj3 albert) or (samokutyayev adj3 aleksandr) or (sarafanov adj3 gennadi) or (satcher adj3 robert) or (savinykh adj3 viktor) or (savitskaya adj3 svetlana) or (schirra adj3 walter) or (schlegel adj3 hans) or (schmitt adj3 harrison) or (schweickart adj3 russell) or (scobee adj3 francis) or (scott adj3 david) or (scott adj3 winston) or (scully-power adj3 (paul or desmond)) or (searfoss adj3 richard) or (seddon adj3 (margaret or rhea)) or (sega adj3 ronald) or (sellers adj3 piers) or

(serebrov adj3 aleksandr) or (serova adj3 yelena) or (sevastyanov adj3 vitali) or (shargin adj3 yuri) or (sharipov adj3 salizhan) or (shatalov adj3 vladimir) or (shaw adj3 brewster) or (shepard adj3 alan) or (shepherd adj3 william) or (shkaplerov adj3 anton) or (shonin adj3 georgi) or (shriver adj3 loren) or (shukor adj3 (sheikh or muszaphar)) or (shuttleworth adj3 richard) or (simonyi adj3 (charles or karoly)) or (skripochka adj3 oleg) or (skvortsov adj3 aleksandr) or (slayton adj3 donald) or (smith adj3 (michael or john)) or (smith adj3 steven) or (solovyev adj3 (anatolyi or vladimir)) or (spring adj3 sherwood) or (springer adj3 robert) or (stafford adj3 thomas) or (stefanyshyn-piper adj3 heidemarie) or (steven adj maclean) or (stewart adj3 robert) or (stott adj3 nicole) or (strekalov adj3 gennadi) or (sturckow adj3 frederick) or (sullivan adj3 kathryn) or (surayev adj3 maksim) or (swanson adj3 steven) or (swigert adj3 john) or (tani adj3 daniel) or (tanner adj3 joseph) or (tarelkin adj3 yevgeni) or (tereshkova adj3 valentina) or (thagard adj3 norman) or (thiele adj3 gerhard) or (thomas adj3 andrew) or (thomas adj3 donald) or (thornton adj3 kathryn) or (thornton adj3 william) or (thuot adj3 pierre) or (tito adj3 (dennis or anthony)) or (titov adj3 gherman) or (titov adj3 vladimir) or (tognini adj3 michel) or (tokarev adj3 valeri) or (treshchov adj3 sergei) or (trinh adj3 (eugene or huu-chau)) or (truly adj3 richard) or (tsibliyev adj3 vasili) or (tyurin adj3 mikhail)).ti,ab,kw.

10. ((usachov adj3 yuri) or ("van hofteni" adj3 james) or (vasyutin adj3 vladimir) or (veach adj3 (charles or lacy)) or (viehbock adj3 franz) or (viktorenko adj3 aleksandr) or (vinogradov adj3 pavel) or (virts adj3 terry) or (vittori adj3 roverto) or vladimir remek or (volk adj3 igor) or (volkov adj3 (sergei or vladislav or alexander)) or (volynov adj3 boris) or (voss adj3 (janice or james)) or (wakata adj3 koichi) or (walheim adj3 rex) or (walker adj3 (charles or mathieson or shannon or david)) or (walter adj3 ulrich) or (walz adj3 carl) or (wang adj3 (yapi or taylor or gun-jin)) or (weber adj3 mary) or (weitz adj3 paul) or (wetherbee adj3 james) or (wheelock adj3 douglas) or (white adj3 edward) or (whitson adj3 peggy) or (wilcutt adj3 terrence) or (williams adj3 (dafydd or rhys or donald or sunita or jeffrey)) or (wilmore adj3 barry) or (wilson adj3 stephanie) or (wiseman adj3 (gregory or reid)) or (wisoff adj3 peter) or (wolf adj3 david) or (worden adj3 alfred) or (yamazaki adj3 naoko) or (yang adj3 liwei) or (yegorov adj3 boris) or (yeliseyev adj3 aleksei) or (yi adj3 (soyeon or "lee so-hyuni")) or (young adj3 john) or (yui adj3 kimiya) or (yurchikhin adj3 fyodor) or (zalyotin adj3 sergei) or (zamka adj3 george) or (zhai adj3 zhigang) or (zhang adj3 xiaoguang) or (zholobov adj3 vitali) or (zudov adj3 vyacheslav)).ti,ab,kw.

11. or/1-10

12. exp Astronauts/

13. exp Space Flight/

14. exp Weightlessness/

15. exp Extraterrestrial Environment/

16. ((soyuz* or apollo* or gemini or "international space station" or saluyt or skylab or shenzhou or voskhod or euromir or NASA or voskhod or tiangong or mir or mercury or shuttle or ISS or ESA or CNSA or NASDA) and (space* or orbit* or station* or mission*)).ti,ab,kf.

17. (astronaut* or cosmonaut*).ti,ab,kf.

18. (space adj5 (flight* or travel* or explor* or outer)).ti,ab,kw.

19. or/1-18

20. exp "Bone and Bones"/

21. exp Bone Diseases/

22. exp Osteogenesis/

23. exp Bone Density/

24. exp Bone Remodeling/

25. (bone* or osseo* or osteo* or skelet* or musculoskelet*).ti,ab,kw.

26. (skeletal or musculoskeletal or tarsal* or metatarsal* or calcaneus or talus or femur or fibula or patella or fibia or humerus or radius or ulna or clavicle or acromion or glenoid or diaphyses or epiphyses or hyoid or sesamoid or cranium or cranial or occipital or basilar or foramen or basicranium or sphenoid or mastoid or petrous or odontoid or parietal or fossa or skull or spenoid or mandible or maxilla or vomer or zygoma or vertebra* or sacrum or rib or ribs or sternum or manubrium or coccyx).ti,ab,kw.

27. or/20-26

28. 19 and 27

2. Quality checklist for full text appraisal

A maximal possible score is 15:

1. Article type: conference abstract (0 points), review (0), primary study (1);
2. A clear reporting of (1 point each, maximum of 7): mission title, flight duration, number of astronauts in a study, time of pre-flight, in-flight and post-flight measurements, type of techniques used;
3. Data was presented in a tabular (1) or graphical (0) form;
4. Data was presented for individual astronauts (1);
5. Measure of data variation (standard deviation, standard error, range) was provided for aggregate outcomes (1);
6. Sex (1) and age (1) of astronauts was reported;
7. Bone mineral density (1), biochemical bone markers (1) and hormone levels (1) were reported.

3. MATLAB code used to fit piece-wise functions to biochemical bone resorption and formation data post-flight.

```
%% monte carlo exponential
clear all; close all;

%% resorptionMarkers
sheet1='Arnaud'; flightDur(1)=14;
sheet2='Smith1998Skylab2'; flightDur(2)=28;
sheet3='Smith1998Skylab3'; flightDur(3)=59;
sheet4='Smith1998Skylab4'; flightDur(4)=84;
sheet5='Smith2005'; flightDur(5)=150;
sheet6='Caillot2000Euromir97'; flightDur(6)=21;
sheet7='decay'; flightDur(7)=100;

%% formationMarkers (pre and post)
sheet8='Arnaud1995'; flightDur(8)=14;
sheet9='Caillot2000'; flightDur(9)=21% 90; %21;
sheet10='Collet1997'; flightDur(10)=28;
sheet11='Grigoriev1999_1'; flightDur(11)=197; % 197;
sheet12='Grigoriev1999_2'; flightDur(12)=208; %208;
sheet13='Smith2005Group2'; flightDur(13)=50; %150;

%% formationMarkers (pre only)
sheet14='Smith2015ARED'; flightDur(14)=173;
sheet15='Smith2015iRED'; flightDur(15)=148;

%% formationMarkers (post only)
sheet16='Grigoriev1999_3'; flightDur(16)=14;
sheet17='Grigoriev1999_4'; flightDur(17)=14;
sheet18='Grigoriev1999_5'; flightDur(18)=14;
sheet19='Smith2005Group1'; flightDur(19)=15;
sheet20='Johnston1977'; flightDur(20)=15;
sheet21='Morukov2005'; flightDur(21)=20;
sheet22='Morukov2014'; flightDur(22)=10;
sheet23='Nicogossian1977'; flightDur(23)=30;
sheet24='Parker1975'; flightDur(24)=15;
sheet25='Yegorov1979'; flightDur(25)=28;

%% resorptionMarkers (pre only)
sheet26='Smith2015ARED'; flightDur(26)=173;
sheet27='Smith2015iRED'; flightDur(27)=148;

%% resorptionMarkers (post only)
sheet28='Smith2005Study1'; flightDur(28)=150;
```

```
sheet29='Morukov2005'; flightDur(29)=20;
sheet30='Morukov2014'; flightDur(30)=30;

%% formationMarker_vsDur
sheet31='decay1'; flightDur(31)=220;
sheet32='decay2'; flightDur(32)=220;

input = sheet31

Dur = flightDur(31);
[num,txt,raw]= xlsread('10-formation_101018.xlsx', input);
% [num,txt,raw]= xlsread('10-formation_101018_cropPost.xlsx', input);
% [num,txt,raw]= xlsread('10-resorption_101018.xlsx', input);

ID = num(:,1);
nr = num(:,5);
xr = num(:,3);
ser = num(:,4);
day = num(:,6);
inPost = num(:,7);

model = '3'; % 1 hill, 2 expo, 3 linear, 4 quadratic, 5-1 piecewise hill, 5-2 piecewise linear
piece = '1' % 1 piecelinear, 2 pieceexpo
%

xr2 = xr(inPost==1)
nr2 = nr(inPost==1)
day2 = day(inPost==1)
ser2 = ser(inPost==1)

xr = xr(inPost==0)
nr = nr(inPost==0)
day = day(inPost==0)
ser = ser(inPost==0)

figure; plot (day, xr, 'o');

% day2= [Dur; day2];
try; daySmooth = [0:0.1:Dur]; catch;  daySmooth = [0:0.1:max(day)]; end
daySmooth2 = [Dur:0.1:250];
% daySmooth2 = [Dur:0.1:max(day2)*1.1];
i=1; go = 0;
while i < 500
   i
   if go == 1; i = 1+i; end; go = 1;
```

```
    try;
        for j = 1:length(xr); newData{i}(j) = mean(normrnd(xr(j),ser(j)*sqrt(nr(j)),[1,nr(j)])); end
%         newData{i}(:) = holder; holder = [];
        parfor j = 1:length(xr); holder(j) = mean(normrnd(xr(j),ser(j)*sqrt(nr(j)),[1,nr(j)])); end
        newData{i}(:) = holder; holder = [];
        try;
%             for j = 1:length(xr2); newData2{i}(j) =
mean(normrnd(xr2(j),ser2(j)*sqrt(nr2(j)),[1,nr2(j)])); end
            parfor j = 1:length(xr2); holder(j) = mean(normrnd(xr2(j),ser2(j)*sqrt(nr2(j)),[1,nr2(j)]));
end
            newData2{i}(:) = holder; holder = [];
        catch e; disp e; end

        figure(1); hold on;
        switch model
            case '1'
                [fitresult{i}, gof{i}] = hillFit(day,  newData{i});
                coef{i} = coeffvalues(fitresult{i});
                aR(i) = coef{i}(1);
                bR(i) = coef{i}(2);
                cR(i) = coef{i}(3);
                plot(daySmooth, aR(i).*(daySmooth.^(bR(i))) ./ ((cR(i).^bR(i))+(daySmooth.^bR(i))) ,
'r');
            case '2'

                [fitresult{i}, gof{i}] = expoFit(day,  newData{i});
                coef{i} = coeffvalues(fitresult{i});
                aR(i) = coef{i}(1);
                bR(i) = -coef{i}(2);
                 plot(daySmooth, aR(i).*exp(-bR(i).*(daySmooth))  , 'r'); hold on;
            case '3' % linear
                [fitresult{i}, gof{i}] = fitline(day,  newData{i})
                coef{i} = coeffvalues(fitresult{i});
                aR(i) = coef{i}(1);
                bR(i) = coef{i}(2);
                plot(daySmooth, aR(i).*daySmooth + bR(i) , 'r');

            case '4' % quadratic
                [fitresult{i}, gof{i}] = fitQuadratic(day,  newData{i})
                coef{i} = coeffvalues(fitresult{i});
                aR(i) = coef{i}(1);
                bR(i) = coef{i}(2);
                cR(i) = coef{i}(3);
                plot(daySmooth,aR(i).*(daySmooth-bR(i)).^2+cR(i) , 'r');

            case '5-1' % piecewise hill
```

```
            [fitresult{i}, gof{i}] = hillFit(day,  newData{i});
            coef{i} = coeffvalues(fitresult{i});
            aR(i) = coef{i}(1);
            bR(i) = coef{i}(2);
            cR(i) = coef{i}(3);
            subplot(121); plot(daySmooth, aR(i).*(daySmooth.^(bR(i))) ./
((cR(i).^bR(i))+(daySmooth.^bR(i))) , 'r'); hold on;

            interc(i) = aR(i).*(Dur.^(bR(i))) ./ ((cR(i).^bR(i))+( Dur.^bR(i)))

            switch piece
               case '1'

                  [fitresult2{i}, gof2{i}] = fitLinConstantInt(day2-Dur,  newData2{i}, interc(i));
                  coef2{i} = coeffvalues(fitresult2{i});
                  dR(i) = coef2{i}(1);
                  eR(i) = interc(i);
                  subplot(122); plot(daySmooth2, dR(i).*(daySmooth2-Dur) +eR(i) , 'r'); hold on;
               case '2'

                  [fitresult2{i}, gof2{i}] = expoFit2(day2-Dur,  newData2{i}, interc(i));
                  coef2{i} = coeffvalues(fitresult2{i});
                  dR(i) = coef2{i}(1);
                  eR(i) = interc(i);
                  subplot(122); plot(daySmooth2, eR(i).*exp(-dR(i).*(daySmooth2-Dur))  , 'r');
hold on;
            end

         case '5-2' % piecewise linear
            [fitresult{i}, gof{i}] = fitline(day,  newData{i})
            coef{i} = coeffvalues(fitresult{i});
            aR(i) = coef{i}(1);
            bR(i) = coef{i}(2);
            subplot(121); plot(daySmooth, aR(i).*daySmooth + bR(i) , 'r'); hold on;
            interc(i) = aR(i).*Dur + bR(i)

            switch piece
               case '1'

                  [fitresult2{i}, gof2{i}] = fitLinConstantInt(day2-Dur,  newData2{i}, interc(i));
                  coef2{i} = coeffvalues(fitresult2{i});
                  dR(i) = coef2{i}(1);
                  eR(i) = interc(i);
                  subplot(122); plot(daySmooth2, dR(i).*(daySmooth2-Dur) +eR(i) , 'r'); hold on;
               case '2'
```

```
                        [fitresult2{i}, gof2{i}] = expoFit2(day2-Dur,  newData2{i}, interc(i));
                        coef2{i} = coeffvalues(fitresult2{i});
                        dR(i) = coef2{i}(1);
                        eR(i) = interc(i);
                        subplot(122); plot(daySmooth2, eR(i).*exp(-dR(i).*(daySmooth2-Dur))  , 'r');
hold on;
                end
            end

            R2(i) = gof{i}.rsquare;

        catch e;
            disp(e)
            go = 0;
        end


end

switch model
    case '1' % hill
        for i = 1:length(daySmooth)
            y_bar(i) =   mean ( aR.*(daySmooth(i).^(bR)) ./ ((cR.^bR)+(daySmooth(i).^bR)));
            y_ciWidth(i) = 1.96*std(aR.*(daySmooth(i).^(bR)) ./ ((cR.^bR)+(daySmooth(i).^bR)));
            y_predWidth(i) = 1.96*(std(aR.*(daySmooth(i).^(bR)) ./
((cR.^bR)+(daySmooth(i).^bR)))*sqrt(length(xr))*sqrt(1+(1/length(xr))));
            y_sd(i) = std(aR.*(daySmooth(i).^(bR)) ./ ((cR.^bR)+(daySmooth(i).^bR)));
            y_ul(i) = y_bar(i)+y_ciWidth(i);
            y_ll(i) = y_bar(i)-y_ciWidth(i);
            y_uPred(i) = y_bar(i)+y_predWidth(i);
            y_lPred(i) = y_bar(i)-y_predWidth(i);
        end

    case '2' % expo
        for i = 1:length(daySmooth)
            y_bar(i) =   mean ( aR.*exp(-bR.*daySmooth(i)));
            y_ciWidth(i) = 1.96*std(aR.*exp(-bR.*daySmooth(i)));
            y_predWidth(i) = 1.96*(std(aR.*exp(-
bR.*daySmooth(i)))*sqrt(length(xr))*sqrt(1+(1/length(xr))));
            y_sd(i) = std(aR.*exp(-bR.*daySmooth(i)));
            y_ul(i) = y_bar(i)+y_ciWidth(i);
            y_ll(i) = y_bar(i)-y_ciWidth(i);
            y_uPred(i) = y_bar(i)+y_predWidth(i);
            y_lPred(i) = y_bar(i)-y_predWidth(i);

        end
```

```
    case '3' %linear
        for i = 1:length(daySmooth)
            y_bar(i) =   mean ( aR.*daySmooth(i) + bR);
            y_ciWidth(i) = 1.96*std(aR.*daySmooth(i) + bR);
            y_predWidth(i) = 1.96*(std(aR.*daySmooth(i) +
bR)*sqrt(length(xr))*sqrt(1+(1/length(xr))));
            y_sd(i) = std(aR.*daySmooth(i) + bR);
            y_ul(i) = y_bar(i)+y_ciWidth(i);
            y_ll(i) = y_bar(i)-y_ciWidth(i);
            y_uPred(i) = y_bar(i)+y_predWidth(i);
            y_lPred(i) = y_bar(i)-y_predWidth(i);

        end

    case '4'  % quadratic
        for i = 1:length(daySmooth)
            y_bar(i) =   mean ( aR.*(daySmooth(i)-bR).^2+cR );
            y_ciWidth(i) = 1.96*std(aR.*(daySmooth(i)-bR).^2+cR );
            y_predWidth(i) = 1.96*(std(aR.*(daySmooth(i)-bR).^2+cR
)*sqrt(length(xr))*sqrt(1+(1/length(xr))));
            y_sd(i) = std(aR.*(daySmooth(i)-bR).^2+cR );
            y_ul(i) = y_bar(i)+y_ciWidth(i);
            y_ll(i) = y_bar(i)-y_ciWidth(i);
            y_uPred(i) = y_bar(i)+y_predWidth(i);
            y_lPred(i) = y_bar(i)-y_predWidth(i);

        end

    case '5-1'  % piecewise
        for i = 1:length(daySmooth)
            y_bar(i) =   trimmean ( aR.*(daySmooth(i).^(bR)) ./ ((cR.^bR)+(daySmooth(i).^bR)),
10);
            y_ciWidth(i) = 1.96*std(aR.*(daySmooth(i).^(bR)) ./ ((cR.^bR)+(daySmooth(i).^bR)));
            y_predWidth(i) = 1.96*(std(aR.*(daySmooth(i).^(bR)) ./
((cR.^bR)+(daySmooth(i).^bR)))*sqrt(length(xr))*sqrt(1+(1/length(xr))));
            y_sd(i) = std(aR.*(daySmooth(i).^(bR)) ./ ((cR.^bR)+(daySmooth(i).^bR)));
            y_ul(i) = y_bar(i)+y_ciWidth(i);
            y_ll(i) = y_bar(i)-y_ciWidth(i);
            y_uPred(i) = y_bar(i)+y_predWidth(i);
            y_lPred(i) = y_bar(i)-y_predWidth(i);
        end

        switch piece
            case '1' % piecewise linear
                for i = 1:length(daySmooth2)
```

```
                y_bar2(i) =   trimmean ( dR.*(daySmooth2(i)-Dur) + eR,10);
                y_ciWidth2(i) = 1.96*std(dR.*(daySmooth2(i)-Dur) + eR);
                y_predWidth2(i) = 1.96*(std(dR.*(daySmooth2(i)-Dur) +
eR)*sqrt(length(xr))*sqrt(1+(1/length(xr))));
                y_sd2(i) = std(dR.*(daySmooth2(i)-Dur) + eR);
                y_ul2(i) = y_bar2(i)+y_ciWidth2(i);
                y_ll2(i) = y_bar2(i)-y_ciWidth2(i);
                y_uPred2(i) = y_bar2(i)+y_predWidth2(i);
                y_lPred2(i) = y_bar2(i)-y_predWidth2(i);

              end

            case '2'       %piecewise expo
%                 eR(i).*exp(-dR(i).*(daySmooth2-min(day2)))
              for i = 1:length(daySmooth2)
                y_bar2(i) =   trimmean ( eR.*exp(-dR.*(daySmooth2(i)-Dur)) ,10);
                y_ciWidth2(i) = 1.96*std( eR.*exp(-dR.*(daySmooth2(i)-Dur)));
                y_predWidth2(i) = 1.96*(std( eR.*exp(-dR.*(daySmooth2(i)-
Dur)))*sqrt(length(xr))*sqrt(1+(1/length(xr))));
                y_sd2(i) = std( eR.*exp(-dR.*(daySmooth2(i)-Dur)));
                y_ul2(i) = y_bar2(i)+y_ciWidth2(i);
                y_ll2(i) = y_bar2(i)-y_ciWidth2(i);
                y_uPred2(i) = y_bar2(i)+y_predWidth2(i);
                y_lPred2(i) = y_bar2(i)-y_predWidth2(i);

              end
          end

          case '5-2'  % piecewise linear
          for i = 1:length(daySmooth)
                 y_bar(i) =   trimmean ( aR.*daySmooth(i) + bR, 10);
            y_ciWidth(i) = 1.96*std(aR.*daySmooth(i) + bR);
            y_predWidth(i) = 1.96*(std(aR.*daySmooth(i) +
bR)*sqrt(length(xr))*sqrt(1+(1/length(xr))));
            y_sd(i) = std(aR.*daySmooth(i) + bR);
            y_ul(i) = y_bar(i)+y_ciWidth(i);
            y_ll(i) = y_bar(i)-y_ciWidth(i);
            y_uPred(i) = y_bar(i)+y_predWidth(i);
            y_lPred(i) = y_bar(i)-y_predWidth(i);
          end

          switch piece
            case '1' % piecewise linear
              for i = 1:length(daySmooth2)
                y_bar2(i) =   trimmean ( dR.*(daySmooth2(i)-Dur) + eR,10);
                y_ciWidth2(i) = 1.96*std(dR.*(daySmooth2(i)-Dur) + eR);
```

```
            y_predWidth2(i) = 1.96*(std(dR.*(daySmooth2(i)-Dur) + 
eR)*sqrt(length(xr))*sqrt(1+(1/length(xr))));
            y_sd2(i) = std(dR.*(daySmooth2(i)-Dur) + eR);
            y_ul2(i) = y_bar2(i)+y_ciWidth2(i);
            y_ll2(i) = y_bar2(i)-y_ciWidth2(i);
            y_uPred2(i) = y_bar2(i)+y_predWidth2(i);
            y_lPred2(i) = y_bar2(i)-y_predWidth2(i);

          end

        case '2'      %piecewise expo
%             eR(i).*exp(-dR(i).*(daySmooth2-min(day2)))
          for i = 1:length(daySmooth2)
            y_bar2(i) =   trimmean ( eR.*exp(-dR.*(daySmooth2(i)-Dur)) ,10);
            y_ciWidth2(i) = 1.96*std( eR.*exp(-dR.*(daySmooth2(i)-Dur)));
            y_predWidth2(i) = 1.96*(std( eR.*exp(-dR.*(daySmooth2(i)-
Dur)))*sqrt(length(xr))*sqrt(1+(1/length(xr))));
            y_sd2(i) = std( eR.*exp(-dR.*(daySmooth2(i)-Dur)));
            y_ul2(i) = y_bar2(i)+y_ciWidth2(i);
            y_ll2(i) = y_bar2(i)-y_ciWidth2(i);
            y_uPred2(i) = y_bar2(i)+y_predWidth2(i);
            y_lPred2(i) = y_bar2(i)-y_predWidth2(i);

          end
      end
end


figure; hold on;
plot(daySmooth, y_bar,'r');
plot(daySmooth, y_ll,'r'); plot(daySmooth, y_ul,'r');
% plot(daySmooth, y_lPred,'r'); plot(daySmooth, y_uPred,'r');

try;
   plot(daySmooth2, y_bar2,'r');
   plot(daySmooth2, y_ll2,'r'); plot(daySmooth2, y_ul2,'r');
   % plot(daySmooth2, y_lPred2,'r'); plot(daySmooth2, y_uPred2,'r');
catch e; disp e; end

nrmod = (((nr/max(nr))*0.9)+0.2)*5;
for j = 1:length(xr);     plot (day(j), xr(j),'ko', 'markerSize', nrmod(j)); end

try;
   nrmod2 = (((nr2/max(nr2))*0.9)+0.2)*5;
   for j = 1:length(xr2);     plot (day2(j), xr2(j),'bo', 'markerSize', nrmod2(j)); end
catch; end
```

```
title(input);
% ylim([-50 inf]);
% ylim([0 200]);

disp(['R2 (median) = ' num2str(median(R2))]);
disp(' ');
disp(['a (median)= ' num2str(median(aR))]);
disp(['a (mean)= ' num2str(mean(aR))]);
disp(['a (std)= ' num2str(std(aR))]);
disp(' ');
disp(['b (median)= ' num2str(median(bR))]);
disp(['b (mean)= ' num2str(mean(bR))]);
disp(['a (std)= ' num2str(std(bR))]);
disp(' ');
try;
disp(['c (median)= ' num2str(median(cR))]);
disp(['c (mean)= ' num2str(mean(cR))]);
disp(['a (std)= ' num2str(std(cR))]); catch; end
disp(' ');
disp(['d (median)= ' num2str(median(dR))]);
disp(['d (mean)= ' num2str(mean(dR))]);
disp(['a (std)= ' num2str(std(dR))]);
disp(' ');
disp(['e (median)= ' num2str(median(eR))]);
disp(['e (mean)= ' num2str(mean(eR))]);
disp(['a (std)= ' num2str(std(eR))]);



switch model
    case '2'
        figure; subplot(121); hist(aR); subplot(122); hist(bR);
end


disp(median(R2));
try;
    figure; hold on;
    subplot(311); hist(aR); subplot(312); hist(bR); subplot(313); hist(cR);
catch; end

function [fitresult, gof] = expoFit(day, xr)

%% Fit: 'untitled fit 1'.
[xData, yData] = prepareCurveData( day, xr );
```

```
% Set up fittype and options.
ft = fittype( 'exp1' );
opts = fitoptions( 'Method', 'NonlinearLeastSquares' );
opts.Display = 'Off';
opts.StartPoint = [100 -0.01];

% opts.Lower = [-0.03 0.01];
% opts.Upper = [0.1 0.04];
% Fit model to data.
[fitresult, gof] = fit( xData, yData, ft, opts );


% opts.StartPoint = [46.0443799088857 0.00518053472785949];

% % Plot fit with data.
% figure( 'Name', 'untitled fit 1' );
% h = plot( fitresult, xData, yData );
% legend( h, 'xr vs. day', 'untitled fit 1', 'Location', 'NorthEast' );
% % Label axes
% xlabel day
% ylabel xr
% grid on

end


function [fitresult, gof] = expoFit2(day, xr, interc)


%% Fit: 'untitled fit 1'.
[xData, yData] = prepareCurveData( day, xr );

func = [ num2str(interc) '*exp(-b*x)'];
% Set up fittype and options.
ft = fittype( func, 'independent', 'x', 'dependent', 'y' );
opts = fitoptions( 'Method', 'NonlinearLeastSquares' );
opts.Display = 'Off';
opts.StartPoint = 0.01;

opts.Lower = [0];
% opts.StartPoint = [46.0443799088857 0.00518053472785949];
opts.Upper = [0.05];

% Fit model to data.
[fitresult, gof] = fit( xData, yData, ft, opts );
```

```
end


function [fitresult, gof] = hillFit(day, xr)

%% Fit: 'untitled fit 1'.
[xData, yData] = prepareCurveData( day, xr );

% Set up fittype and options.
ft = fittype( 'a*(x^(b)) / ((c^b)+(x^b))', 'independent', 'x', 'dependent', 'y' );
opts = fitoptions( 'Method', 'NonlinearLeastSquares' );
opts.Display = 'Off';
opts.StartPoint = [120 0.1 10];

opts.Lower = [0 0.01 2];
% opts.StartPoint = [46.0443799088857 0.00518053472785949];
opts.Upper = [200 1 50];
% Fit model to data.
[fitresult, gof] = fit( xData, yData, ft, opts );


end


function [fitresult, gof] = fitline(day, xr)

%% Fit: 'untitled fit 1'.
[xData, yData] = prepareCurveData( day, xr );

% Set up fittype and options.
ft = fittype( 'poly1' );

% Fit model to data.
[fitresult, gof] = fit( xData, yData, ft );

end


function [fitresult, gof] = fitQuadratic(day, xr)

%% Fit: 'untitled fit 1'.
[xData, yData] = prepareCurveData( day, xr );

% Set up fittype and options.
ft = fittype( 'a*(x-b)^2+c', 'independent', 'x', 'dependent', 'y' );
```

```
opts = fitoptions( 'Method', 'NonlinearLeastSquares' );
opts.Display = 'Off';
opts.StartPoint = [-0.1 25 80];

% Fit model to data.
[fitresult, gof] = fit( xData, yData, ft, opts );

end


function [fitresult, gof] = fitLinConstantInt(day, xr, interc)

%% Fit: 'untitled fit 1'.
[xData, yData] = prepareCurveData( day, xr );

func = ['b*x+' num2str(interc)];

% Set up fittype and options.
ft = fittype( func, 'independent', 'x', 'dependent', 'y' );
opts = fitoptions( 'Method', 'NonlinearLeastSquares' );
opts.Display = 'Off';
opts.StartPoint = -0.296852663545566;

% Fit model to data.
[fitresult, gof] = fit( xData, yData, ft, opts );
end
```
